# Improved Efficiency and Robustness in qPCR and Multiplex End-Point PCR by Twisted Intercalating Nucleic Acid Modified Primers

**DOI:** 10.1371/journal.pone.0038451

**Published:** 2012-06-06

**Authors:** Uffe Vest Schneider, Nikolaj Dam Mikkelsen, Anja Lindqvist, Limei Meng Okkels, Nina Jøhnk, Gorm Lisby

**Affiliations:** QuantiBact A/S, Hvidovre, Denmark; Instituto Butantan, Laboratório Especial de Toxinologia Aplicada, Brazil

## Abstract

We introduce quantitative polymerase chain reaction (qPCR) primers and multiplex end-point PCR primers modified by the addition of a single *ortho*-Twisted Intercalating Nucleic Acid (*o*-TINA) molecule at the 5′-end. In qPCR, the 5′-*o*-TINA modified primers allow for a qPCR efficiency of 100% at significantly stressed reaction conditions, increasing the robustness of qPCR assays compared to unmodified primers. In samples spiked with genomic DNA, 5′-*o*-TINA modified primers improve the robustness by increased sensitivity and specificity compared to unmodified DNA primers. In unspiked samples, replacement of unmodified DNA primers with 5′-*o*-TINA modified primers permits an increased qPCR stringency. Compared to unmodified DNA primers, this allows for a qPCR efficiency of 100% at lowered primer concentrations and at increased annealing temperatures with unaltered cross-reactivity for primers with single nucleobase mismatches. In a previously published octaplex end-point PCR targeting diarrheagenic *Escherichia coli*, application of 5′-*o*-TINA modified primers allows for a further reduction (>45% or approximately one hour) in overall PCR program length, while sustaining the amplification and analytical sensitivity for all targets in crude bacterial lysates. For all crude bacterial lysates, 5′-*o*-TINA modified primers permit a substantial increase in PCR stringency in terms of lower primer concentrations and higher annealing temperatures for all eight targets. Additionally, crude bacterial lysates spiked with human genomic DNA show lesser formation of non-target amplicons implying increased robustness. Thus, 5′-*o*-TINA modified primers are advantageous in PCR assays, where one or more primer pairs are required to perform at stressed reaction conditions.

## Introduction

Optimization of a conventional quantitative polymerase chain reaction (qPCR) assay requires consideration of a number of different parameters such as primer design, primer concentration, buffer composition, choice of polymerase and assay temperature profile. Another fundamental challenge in the development of qPCR assays for clinical diagnostics, e.g. for infectious diseases, is the frequent need for highly multiplexed assays to cover all relevant targets [1]. To ease the design of these multiplex qPCR assays, it is beneficial if the primer concentrations can be reduced without decreasing the qPCR efficiency in order to diminish the likelihood of primer cross-reactivity and amplification of non-target nucleotide sequences [2], [3]. Likewise, the ability to increase the annealing temperature or decrease the primer length without negative impact on the qPCR efficiency is desirable in order to diminish cross-reactivity of the qPCR primers and subsequent problems with assay specificity [2], [3]. Additionally, shortening the primer lengths not only increases assay specificity, but also eases primer design for clinical relevant targets by expanding the range of possible target regions. These benefits are especially important for targets with high mutation rates and when designing multiplexed qPCR assays.

**Figure 1:**
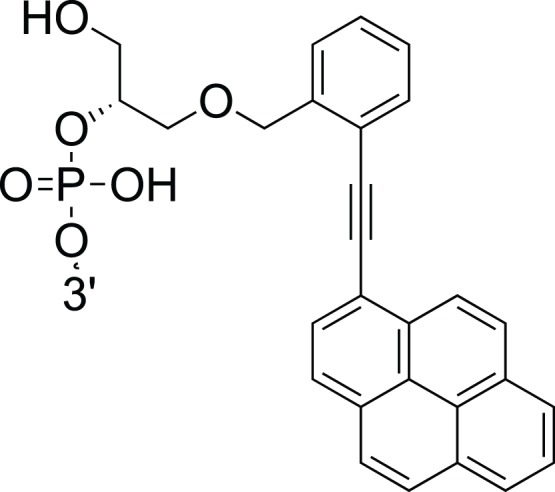
*Ortho*-TINA (*o*-TINA) modification attached to the 5′-end of the primer sequence.

Twisted Intercalating Nucleic Acid (TINA) is a novel group of nucleic acid intercalating molecules (Figure 1) [4], [5]. We have previously shown that *ortho*-TINA (*o*-TINA) modified oligonucleotides can improve the analytical sensitivity of a hybridization capture assay without increasing the cross-reactivity [6]. For optimal thermal stabilization of antiparallel duplex helixes, the *o*-TINA molecules are added by covalent linkage at the 5′- and/or 3′- terminal ends of oligonucleotide sequences [6]. This is different from artificial nucleic acids and nucleic acid mimics, such as Locked Nucleic Acid (LNA) and Peptide Nucleic Acid (PNA) which are placed internally as nucleotide substitutions in the oligonucleotide sequence, and which are well known to inhibit the DNA polymerase when placed in PCR primers [7], [8]. We have tested 5′-*o*-TINA modified oligonucleotides as PCR primers, since the observed improved analytical sensitivity in oligonucleotide hybridization could potentially translate into improved efficacy of the PCR reaction.

Since the introduction of PCR by K. B. Mullis et al. [9], a number of studies have introduced different modifications to the PCR that can increase PCR efficiency [10]–[16]. To assess the impact of different variables on PCR efficiency, qPCR is well suited, as the PCR efficiency and product yield can be monitored in real-time. An enhancement of the qPCR efficiency is either demonstrated as an increase in qPCR efficiency for an assay under suboptimal conditions [10]–[12], [14] or as the amount of added stress (reduced primer concentration, reduced annealing time, decreased buffer salt concentrations or increased annealing temperature) that will still allow for 100% qPCR efficiency for a qPCR assay performing at 100% efficiency before the added stress [13]. A number of small molecules (e.g. magnesium, low molecular amides and formamide) have been shown to improve qPCR efficiency under suboptimal qPCR conditions by globally decreasing buffer stringency - but at the cost of target specificity [10]–[12], [15], [16]. Local improvements in primer annealing promise enhanced qPCR efficiency and robustness, without the negative influence on assay specificity.

To identify the potential effects of 5′-*o*-TINA modifications of PCR primers, we used a qPCR assay targeting the *porA* pseudogene from *Neisseria gonorrhoeae* (*N. gonorrhoeae*) as a model system [17]. Reaction conditions were optimized to allow for optimal qPCR efficiency by unmodified DNA primers. The optimal qPCR conditions were identified as the combination of the maximum annealing temperature (T*a*) and the minimum primer concentration (C*primers*) allowing for 100% qPCR efficiency. Based upon these optimal reaction conditions for unmodified DNA primers, the qPCR assay was further incrementally stressed by increasing the T*a* and decreasing the C*primers*. The resulting qPCR efficiency under these stressed conditions were tested for both the unmodified DNA primers and the 5′-*o*-TINA modified primers and are reported as part of the present study. To ensure the applicability of our findings to PCR assays in general, we have tested the impact of 5′-*o*-TINA modified primers on a previously published octaplex end-point PCR assay, which is routinely used in clinical microbiology for identification of human diarrheagenic *Escherichia coli* (*E. coli*) and *Shigella* species [18].

## Materials and Methods

### Oligonucleotides

All oligonucleotides for the *N*. *gonorrhoeae* qPCR assay were purchased from DNA Technology A/S (Risskov, Denmark) except the unmodified match forward primer, which was purchased from Eurofins (Ebersberg, Germany) on a 0.2 µmol synthesis scale. The oligonucleotides for the diarrheagenic *E*. *coli* octaplex end-point PCR assay were purchased from Eurofins (Ebersberg, Germany) on a 0.2 µmol synthesis scale. All primers were synthesized on an ABI-3900 with reverse phase high performance liquid chromatography (RP-HPLC) purification and a final quality control by mass spectrometry analysis before lyophilization. The *ortho*-TINA amidite, used for the oligonucleotide synthesis, was synthesized as reported in [6] and 5′-*o*-TINA modified primers for research purposes are available through Eurofins (Ebersberg, Germany).

The forward and reverse primer sequences used for amplification of the *porA* pseudogene from *N. gonorrhoeae* were 5′- CCG GAA CTG GTT TCA TCT G -3′ and 5′- GTT TCA GCG GCA GCA TTC A -3′, respectively. The sequences of the 5′-*o*-TINA (Z) modified primers were 5′- ZCC GGA ACT GGT TTC ATC TG -3′ and 5′- ZGT TTC AGC GGC AGC ATT CA -3′. Primer sequences containing single nucleotide mismatches are included in Table S1. The primer sequences used for the diarrheagenic *E. coli* octaplex PCR assay were identical to the sequences published by Brandal LT et al [18] and the sequences are listed below as 5′-*o*-TINA (Z) modified primers. The sequences for the unmodified primers are identical, but without the Z modification. The primer sequences were: *aggR* forward 5′- ZGT ATA CAC AAA AGA AGG AAG C -3′, *aggR* reverse 5′- ZAC AGA ATC GTC AGC ATC AGC -3′, *eae* forward 5′- ZTC AAT GCA GTT CCG TTA TCA GTT -3′, *eae* reverse 5′- ZGT AAA GTC CGT TAC CCC AAC CTG -3′, *elt* forward 5′- ZTC TCT ATG TGC ATA CGG AGC -3′, *elt* reverse 5′- ZCC ATA CTG ATT GCC GCA AT -3′, *estAh* forward 5′- ZAT TTT TCT TTC TGT ATT GTC TT -3′, *estAh* reverse 5′- ZCA CCC GGT ACA AGC AGG ATT -3′, *ipaH* forward 5′- ZGT TCC TTG ACC GCC TTT CCG ATA CCG TC -3′, *ipaH* reverse 5′- ZGC CGG TCA GCC ACC CTC TGA GAG TAC -3′, *rrs* forward 5′- ZCC CCC TGG ACG AAG ACT GAC -3′, *rrs* reverse 5′- ZAC CGC TGG CAA CAA AGG ATA -3′, *stx1* forward 5′- ZAA ATC GCC ATT CGT TGA CTA CTT CT -3′, *stx1* reverse 5′- ZTG CCA TTC TGG CAA CTC GCG ATG CA -3′, *stx2* forward 5′- ZCA GTC GTC ACT CAC TGG TTT CAT CA -3′ and *stx2* reverse 5′- ZGG ATA TTC TCC CCA CTC TGA CAC C -3′. The molecular weight of an *o-*TINA monomer inserted into an oligonucleotide with all protecting groups removed is 467.4 g/mol with an extinction coefficient of 24,090 at 260 nm.

The primers were delivered as lyophilized primers and redissolved in double-distilled water to a stock concentration of 100 µM (based upon the reported UV measurements from the oligonucleotide supplier) and left overnight at 4°C before use. Primer concentrations were verified by triplicate UV-absorbance measurements for each primer on a NanoDrop™ 1000 (Thermo Scientific Inc., Wilmington, NC, USA). The absorbance added by the 5′-*o*-TINA modification was not taken into account for the determination of primer concentrations. For the diarrheagenic *E. coli* octaplex PCR assay, a 10-fold standard mix of either all unmodified or 5′-*o*-TINA modified primers was made based upon the UV-absorbance measurements from the NanoDrop™ 1000. Both standard mixes contained two µM of each primer, except for the *estAh* primer pair for which four µM of each primer was used. For the *N. gonorrhoeae* qPCR assay, the average primer concentration of the 19 unmodified primers from DNA Technology A/S was 106.1 µM (range 86.9 µM to 134.0 µM), whereas the average primer concentration of the 20 5′-*o*-TINA modified primers from DNA Technology A/S was 96.2 µM (range 81.1 µM to 110.8 µM). The average concentration of the match unmodified forward primer for the *N. gonorrhoeae* qPCR assay from Eurofins was 125.4 µM. The triplicate measurements on the NanoDrop™ 1000 differed in average by 1.0% (range 0.1% to 2.5%). The purity of the redissolved primers for the *N. gonorrhoeae* qPCR assay was verified by Liquid chromatography – mass spectrometry (LC-MS) by DNA Technology A/S (Risskov, Denmark). LC-MS analyses for the *N. gonorrhoeae* qPCR assay primers were run on a Thermo LCQ Fleet (Thermo Scientific Inc., Wilmington, NC, USA) in negative mode and calculations were done in Promass for Xcalibur (Novatia, LCC, Monmouth Junction, NJ). For LC-MS, an ACE-3 C18-300, 50×2.1 mm column (Advanced Chromatography Technologies, Aberdeen, United Kingdom) was used with the analytical conditions of 50°C; A gradient from 0-D% B over 20 minutes, A being water; 10 µM EDTA; 1% HFIPA; 0.1% DIEA, B being water: acetonitrile (35∶65); 10 µM EDTA; 1% HFIPA; 0.1% DIEA and D being 25 for unmodified or 50 for 5′-*o*-TINA modified primers. The LC-MS indicates equal purity of unmodified and 5′-*o*-TINA modified primers for the *N. gonorrhoeae* qPCR assay. To avoid introduction of bias by the correction of concentrations without taking the absorbance added by the 5′-*o-*TINA modification into account, we used the primers for the *N. gonorrhoeae* qPCR assay as delivered from the suppliers. This was done as all match unmodified primers and almost all mismatch unmodified primers were higher concentrated and with similar purity compared to the 5′-*o*-TINA modified primers and thereby would favor the efficiency of the unmodified qPCR compared to the reactions, where 5′-*o-*TINA modified primers were applied. Data for all primers for the *N. gonorrhoeae* qPCR assay are included in Table S1.

### Target DNA

As *N. gonorrhoeae* target, we used *N. gonorrhoeae* quantitated bacterial DNA (cat.no. 08-924-000, Advanced Biotechnologies Inc., Columbia MD, USA). For all experiments, we used lot number A0705 containing 15,000 copies/µL determined by qPCR targeting the B protein gene of *N. gonorrhoeae* by the supplier. All target dilutions were done with double-distilled water, on ice immediately before use. The nucleotide sequence of the *N. gonorrhoeae porA* pseudogene is included as Figure S1 based on *N. gonorrhoeae* NCCP11945, GenBank sequence CP001050.1, from nucleotide 736237 to 737148 (912 nucleotides) equaling locus tag NGK 0907 and protein ID ACF29586.1.

As target material for the diarrheagenic *E. coli* multiplex PCR, we used eight clinical *E. coli* strains entailing one or more virulence genes with the *rrs* gene (encoding 16S rRNA) as internal control. The clinical strains were provided by Alice Friis-Møller (Department of Clinical Microbiology, University Hospital of Hvidovre, Copenhagen, Denmark). The clinical strains were identified according to Standard Operating Procedures and are presented by name and virulence factor(s) in brackets: fr1368 (*ipaH*), 55989 (*aggR*), D2259 (*estAh*), D2260 (*elt*), D2168 (*estAh, elt*), D2432 (*stx2*), D3522 (*stx1*), D2188 (*eae, stx1, stx2*). Bacterial DNA was extracted after suspension of approximately half a plated bacterial colony in 100 µL of double distilled water by boiling for 15 minutes and centrifugation at 14,500 rpm for one minute in an Eppendorf MiniSpin plus (Eppendorf Nordic Aps, Hørsholm, Denmark). All target dilutions were done with double-distilled water, on ice immediately before use.

### Preparation of Genomic DNA for Spiking Experiments

Human genomic DNA was purified from the human hepatoma cell line Huh-7.5 [19] provided by Jens Bukh (University of Copenhagen, Denmark) or the leukocyte fraction from whole blood discarded for clinical use provided by the Department of Clinical Immunology - Blood Bank (Rigshospitalet, Denmark) using NucleoBond® AXG columns and the NucleoBond® buffer set IV with addition of buffer G1 (Macherey-Nagel GmbH, Düren, Germany). The protocol was taken from “Genomic DNA and total RNA purification protocols” of December 2010, revision 4 (Macherey-Nagel GmbH, Düren, Germany) following the “Protocol for NucleoBond® CB 500” for “Isolation of genomic DNA from blood and cell cultures”. Eluation was done in double-distilled water. The concentration of dsDNA (dsDNA-50) was measured in triplicate on the NanoDrop™ 1000 (Thermo Fisher Scientific Inc., Wilmington, USA) and the purified DNA was stored at –20°C until use. The concentration of dsDNA from the Huh-7.5 cell line was 927 ng/µL (range 921 ng/µL to 933 ng/µL) with a purity in terms of A_260_/A_280_ of 1.85 (range 1.78 to 1.91), whereas the DNA isolated from whole blood leukocytes had a concentration of 254 ng/µL (range 244 ng/µL to 262 ng/µL) with an A_260_/A_280_ purity of 1.88 (range 1.85 to 1.90).

Genomic DNA from the *E. coli* ATCC 25922 strain was isolated from bacteria harvested in the exponential growth phase using NucleoBond® AXG columns and the NucleoBond® buffer set III (Macherey-Nagel GmbH, Düren, Germany). The protocol was based on “Genomic DNA and total RNA purification protocols”, December 2010, revision 4 (Macherey-Nagel GmbH, Düren, Germany) following the protocol for “Isolation of genomic DNA from bacteria” with the following recommended modifications. For cell disruption 10 mg of lysozyme was added and incubation time was set to one hour. Eluation was done in double-distilled water. The concentration of dsDNA was measured to 1,098 ng/µL on the NanoDrop™ 1000 (Thermo Fisher Scientific Inc., Wilmington, USA) with an A_260_/A_280_ of 1.94. dsDNA was stored at −20°C until use.

### Quantitative PCR Protocol

qPCR was performed in a 10 µL reaction volume using in-house 1x Euro-Optima buffer (10.4 mM Tris-HCl, 56.8 mM Trizma-base, 16.1 mM (NH_4_)_2_SO_4_, 30 mM NaCl, 0.01% Tween80), 3 mM MgCl_2_, 0.08% Bovine Serum Albumine (non-acetylated), 1×SYBR Green I (cat.no. 11988131001, Roche Diagnostics A/S, Hvidovre, Denmark), 0.2 mM of each dNTP (a mixture of dTTP and dUTP was applied as 0.066 mM dTTP and 0.133 mM dUTP), 0.25 Unit of Uracil DNA Glycosylase (cat.no. EN0361, Fermentas GmbH, St. Leon-Rot, Germany), 1 Unit KAPA2G Robust HS (KapaBiosystems, Cape Town, South Africa) in an Abgene® SuperPlate™ 96-well PCR plate (AB gene, Epsom, United Kingdom) sealed with Optically clear, adhesive Microseal® ‘B’ Film (BioRad Laboratories, Copenhagen, Denmark). All qPCR setups were performed manually on ice.

qPCR was performed on the CFX96™ Real-Time System (BioRad Laboratories, Copenhagen, Denmark) utilizing the following cycling conditions: UNG treatment for 10 minutes at 40°C; UNG inactivation and Hot-Start polymerase activation for 10 minutes at 95°C; 45 cycles of denaturation at 95°C for 10 seconds; annealing at different temperatures for 5 seconds; and elongation at 72°C for 5 seconds and plate read. A melting curve profile was collected consisting of 10 seconds at 95°C and a melt curve from 65°C to 90°C with 0.5°C increments per 5 seconds and the samples were cooled for 10 minutes at 10°C. The length of the qPCR amplicon was 102 base pairs (bp).

The primer concentrations used in the experiments were 50, 75, 100, 150, 200, 300, 400, 600 and 800 nM of each primer. The primer “chessboard titration” included the combinations of 100, 200 and 400 nM of each primer. For efficiency curves, we used a three-fold dilution series in triplicates starting from 10,000 copies/well down to 14 copies/well and a negative control (double-distilled water) in triplicate. In all other experiments, the quantification cycle (Cq, previously known as threshold cycle, Ct) values were determined using triplicate measurements of 1,000 copies of target per well with negative control in triplicate utilizing the highest relevant primer concentration. Spiked samples were either added as 10 ng or 100 ng of genomic DNA from *E. coli* ATCC 25922 or the Huh-7.5 cell line per well, which was also added to the negative controls. Annealing temperatures were either 58.0, 60.0, 62.0, 64.0, 66.0, 68.0, 70.0 or 72.0°C. Frequently used annealing temperatures were 60.0°C for unmodified DNA primers and 66.0°C for 5′-*o*-TINA modified primers or a temperature gradient of 60.0 (used for negative controls), 61.0, 63.0, 65.9, 69.5, 72.5, 74.2 and 75.0. In experiments with different annealing times, the annealing time was set to 5, 10 and 30 seconds at 66.0 or 70.0°C to evaluate the influence of annealing time on qPCR efficiency. Specifications of reaction conditions for each experiment can be found in the figure legends and the supplementary figure files.

### End-point Multiplex PCR Protocol and Gel Electrophoresis

The octaplex end-point PCR targeting diarrheagenic *E. coli* was performed in a 50 µL reaction volume in an Eppendorf twin.tec 96-well PCR plate (Eppendorf Nordic Aps, Hørsholm, Denmark) sealed with Optically clear, adhesive Microseal® ‘B’ Film (BioRad Laboratories, Copenhagen, Denmark). 1x Qiagen Multiplex PCR Master Mix (Qiagen Denmark, København Ø, Denmark) was mixed with a primer mix of either unmodified primers or 5′-*o*-TINA modified primers to a final concentration of 200 nM of each primer, except for the *estAh* primer pair for which a final concentration of 400 nM of each primer was used as published by Brandal LT et al [18]. Two µL of boiled bacterial supernatant was added as the maximal target concentration and lower target concentrations were prepared as 10-fold dilution series to a 10,000 dilution of the maximal target concentration and a negative control (double-distilled water). All PCR setups were performed manually on ice.

The octaplex PCR was performed on a SensoQuest Labcycler (SensoQuest GmbH, Göttingen, Germany) utilizing the following cycling conditions: Hot-Start polymerase activation for 15 minutes at 95°C; 30 cycles of denaturation at 94°C for 30 seconds; annealing at 57°C for 30 seconds; and elongation at 72°C for 30 seconds and a final extension step at 72°C for 10 minutes. In the published PCR program from Brandal LT et al. [18], the applied annealing time and elongation time is 90 seconds each. The lengths of the PCR amplicons were 190 bp for *estAh*, 254 bp for *aggR*, 283 bp for *stx2*, 322 bp for *elt*, 370 bp for *stx1*, 401 bp for *rrs*, 482 bp for *eae* and 619 bp for *ipaH*. Amplicons were visualised on 3% agarose gel stained by 1x Gel Red (Biotium Inc, Hayward, CA, USA) with GeneRuler 100 bp Plus DNA Ladder (Fermentas GmbH, St. Leon-Rot, Germany). Pictures were taken on an UV-table with a fixed illumination time of 0.120 seconds.

For evaluation of the octaplex PCR in an *E. coli* DNA free PCR buffer, we used our in-house 1x Euro-Optima buffer system as described for the *N. gonorrhoeae* qPCR assay with unaltered PCR program except for an annealing temperature of 60°C in a 50 µL reaction volume. For crude bacterial lysates spiked with human genomic DNA, we used one µg of human genomic DNA isolated from leukocytes per well. Evaluation of different primer concentrations was done by dilution of the two primer standard mixes containing either unmodified primers or 5′-*o*-TINA modified primers. All dilutions of primer mixes contained equal amounts of each primer, except for the *estAh* primer pair for which the double concentration of each primer was used. Final primer concentrations of 50, 100, 150, 200, 300 and 400 nM of each primer (double for *estAh* primers) were tested for a crude bacterial lysate from strain D2259, a 10-fold dilution of strain D2168 and D3522 and a 100-fold dilution of strain 55989 and D2260. Final primer concentrations of 25, 50, 100, 150, 200 and 300 nM of each primer (double for *estAh* primers) were tested for a 10-fold dilution of strain D2432 and D2188, whereas final primer concentrations of 12.5, 25, 50, 100, 150 and 200 nM of each primer (double for *estAh* primers) were tested for a 100-fold dilution of strain fr1368. For evaluation of the annealing temperature, a temperature gradient of 55.0, 56.6, 57.9, 59.4, 60.8, 62.3, 63.7, 65.2, 66.6, 68.1, 69.5 and 71.0°C was tested for a crude bacterial lysate from strain D2168 and a 10-fold dilution of strain 55989, whereas a temperature gradient of 60.0, 61.5, 62.9, 64.4, 65.8, 67.3, 68.7, 70.2, 71.6, 73.1, 74.5 and 76°C was tested for a 10-fold dilution of strain D2188. As the PCR primers for the *ipaH* gene worked extremely well, a temperature gradient of 65.0, 66.5, 67.9, 69.4, 70.8, 72.3, 73.7, 75.2, 76.6, 78.1, 79.5 and 81.0°C for 60 seconds and with the omission of the 30 seconds of elongation at 72°C was tested for a 100-fold dilution of strain fr1368.

### Data Analysis

For data analysis of the *N. gonorrhoeae* qPCR assay we used the Bio-Rad CFX Manager software, Version 1.1. Raw data were baseline subtracted with curve fit and the single threshold method was used for Cq determinations. The single threshold defined by the software was checked for each plate to be in the middle part of the exponential phase of all amplification curves, and if necessary corrected to the midmost part of the exponential phase. Inter-assay normalization of the Cq determinations were based on triplicate measurement of a control, holding 800 nM of each unmodified primer at 61°C. For each experiment with inter-assay normalization, the threshold was adjusted to be within the middle part of the exponential phase, allowing a mean Cq of 25.11 for the control with a standard deviation (SD) of 0.1 for the experimental controls on each of the four inter-assay normalized plates.

qPCR efficiency was calculated as 10^−1/slope^–1 with the logarithm of the template concentration on the x axis and the Cq plotted on the y axis. A qPCR efficiency of 100% thereby indicates that the amount of PCR product doubles with each cycle.

The melting curves for all samples were checked prior to data analysis. Samples with changes in melting point or major changes in the melting curve profile were excluded from the data analysis. This was done for all samples, except for the experiments with spiked samples, as the visualization of melting curves changes is necessary for the evaluation of these experiments. Negative control melting curves were analyzed to rule out contamination. Exclusion of outliers was held to a minimum, and was only done if single reactions in the triplicate measurements differed significantly (defined as mean +/−1.96×SD). An asterisk in the supplementary data files indicates these excluded outliers. Melting points were defined as the peak of the first derivative. The melting point was a half to one °C higher for all qPCR amplicons modified by 5′-*o-*TINA molecules compared to unmodified DNA amplicons.

## Results

### 5′-*o*-TINA Modified Primers Improved qPCR Efficiency

**Figure 2:**
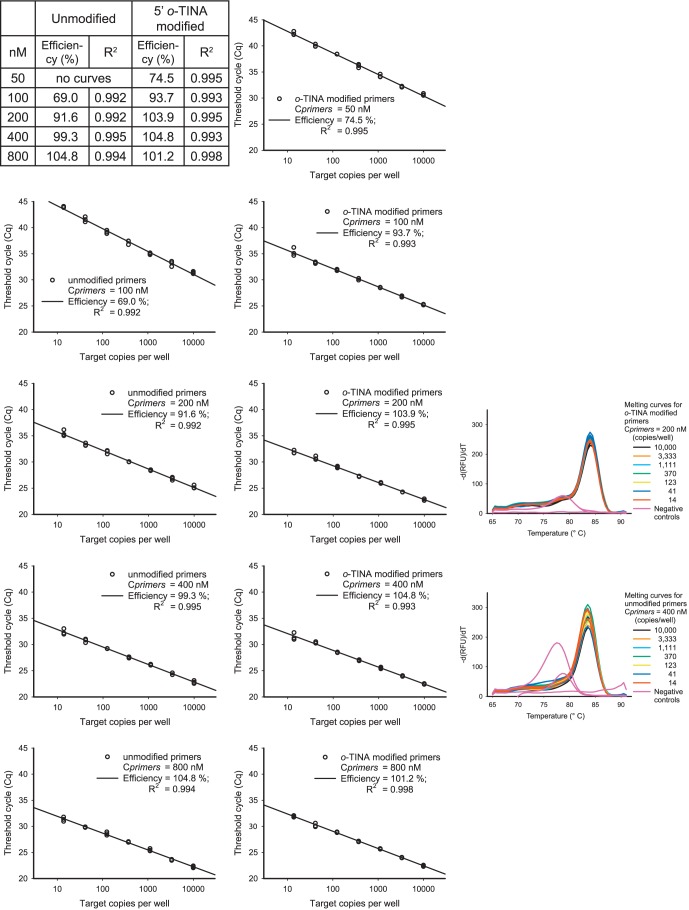
Efficiency curves for unmodified and 5′-*o*-TINA modified primers. All experiments were conducted at an annealing temperature (T*a*) of 66.0°C and primer concentrations (C*primers*) of unmodified and *o*-TINA modified primers were compared on the same plate. The melting curves corresponding to the amplification curves used for efficiency curve determination are included for the lowest C*primers* that allows an efficiency of 100% for unmodified and 5′-*o*-TINA modified primers.

**Figure 3:**
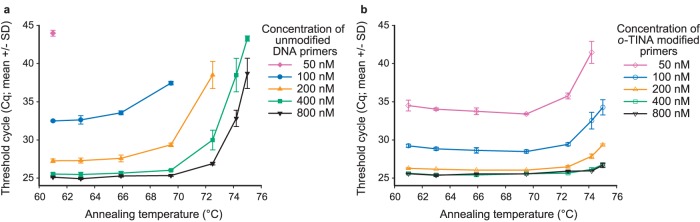
Comparison of unmodified and 5′-*o*-TINA modified primer concentrations on a temperature gradient. (a) Unmodified DNA primer concentrations from 50 nM (⧫) to 800 nM (▾). (b) 5′-*o*-TINA modified primer concentrations from 50 nM (**◊**) to 800 nM (∇). Each threshold cycle (Cq) determination is presented as mean +/− standard deviation (SD) established by triplicate measurements with 1000 copies per well of target. Inter-plate normalization was based on triplicate measurements using 800 nM of unmodified primers and an annealing temperature of 61.0°C (mean Cq for normalization was 25.11 with a SD of 0.1 on each plate).

The unmodified DNA primers were found to allow for a qPCR efficiency of 100%, when a C*primers* of 400 nM or more was used at a T*a* of 66°C or less (Figure 2). A similar (100%) qPCR efficiency at 66°C was obtained with a 5′-*o*-TINA modified C*primers* of 200 nM or more (Figure 2). For both unmodified and 5′-*o*-TINA modified primers, the qPCR efficiency decreased equally as C*primers* were further incrementally decreased, as expected (Figure 2). To address the effect of different C*primers* on the quantification cycle threshold (Cq) [20], we compared unmodified and 5′-*o*-TINA modified C*primers* in the range 50 to 800 nM and a T*a* from 58 to 72°C with 1000 copies of target per reaction (Figure S2). At all C*primers* and T*a*, the Cq of 5′-*o*-TINA modified primers was similar to or lower compared to unmodified primers. Cq indisputably increased at lower C*primers* and increasing T*a* for both unmodified and 5′-*o*-TINA modified primers. The difference in Cq between the unmodified primers and the 5′-*o*-TINA modified primers indisputably increased in favor of the 5′ *o*-TINA modified primers as T*a* was increased towards 72°C, e.g. at 68°C, a DNA C*primers* of 800 nM reached the minimal Cq level, whereas a 5′-*o*-TINA C*primers* of 300 nM or higher reached the same Cq (Figure S2). As the T*a* increased from 58°C towards 72°C, we observed that the minimal Cq level was lowered. This was caused by a decrease in Cq threshold level, since the background signals of the assay decreased by increasing T*a* and thereby increased the signal-to-noise ratio of the assay (Figure S2). We used temperature gradient experiments with inter-assay normalization to evaluate the effect of T*a* on Cq at different C*primers* for both unmodified and 5′-*o*-TINA modified primers (Figure 3). For unmodified primers at C*primers* of 400 nM or more, the minimal Cq was observed at a T*a* below 69°C (Figure 3a). For 5′-*o*-TINA modified primers, a similar Cq was observed at C*primers* of 200 nM or more and at a T*a* below 72°C (Figure 3b). We conclude that 5′-*o*-TINA modified primers compared to unmodified DNA primers sustain a qPCR efficiency of 100% at significantly lower C*primers*, higher T*a* and combinations of both.

### No Difference in qPCR Specificity between Unmodified and 5′-*o*-TINA Modified Primers

To compare the specificity (potential for cross-reactivity) of unmodified DNA and 5′-*o*-TINA modified qPCR primers with single nucleotide mismatches, we applied a T*a* for each C*primers* that allowed for a qPCR efficiency of 100% for match primers. For each C*primers*, it was demonstrated that an increase in T*a* would reduce the qPCR efficiency below 100%, implying that the assay was performed at the most stringent conditions that allowed for a qPCR efficiency of 100% with the match primers (Table S2). Single nucleotide mismatches were introduced in both primers at 5′-, central and 3′- positions in the primers to evaluate the effect of the 5′- terminally placed *o*-TINA molecule on the mismatch discrimination of the primers. The changes in Cq observed by introduction of single nucleotide mismatches in the primers were equal for both unmodified and 5′-*o*-TINA modified primers (Table S2 and Table S3). As expected, Cq increased significantly, when single nucleotide mismatches were introduced in the 3′- part of the primers and diminished towards the 5′- end of the primers (Table S2). As expected, the increase in Cq was enlarged, when the qPCR efficiency for match primers dropped below 100%. All C*primer*s for unmodified and 5′-*o*-TINA modified primers with single nucleotide mismatches were found to increase the Cq equally (Table S2 and Table S3). In conclusion, unmodified and 5′-*o*-TINA modified primers equally impacted the discrimination of single nucleotide mismatches located within the primer sequences.

### 5′-*o*-TINA Modified Primers Increased the Robustness When qPCR Samples were Spiked with Human or Bacterial Genomic DNA

**Figure 4:**
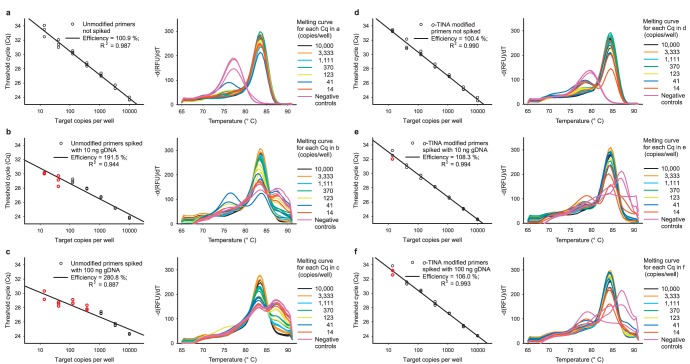
Comparison of primers in samples spiked with human genomic DNA (gDNA). (a-c) unmodified DNA primers and (d-f) 5′-*o*-TINA modified primers. Each ○ on the efficiency curves represents one threshold cycle (Cq) determination on an amplification curve with a corresponding melting curve, reported as the first derivative. Cq determinations highlighted in red would normally have been excluded based on the amplification curve and melting curve evaluation. (a, d) Unspiked samples. (b, e) All samples and negative controls spiked with 10 ng gDNA. (c, f) All samples and negative controls spiked with 100 ng gDNA. A uniform primer concentration of 200 nM was used in all samples and negative controls. The annealing temperatures for unmodified and *o-*TINA primers were 60.0°C and 66.0°C, respectively.

As 5′-*o*-TINA modified primers allow for higher T*a* and lower C*primers* in a qPCR assay without compromising the cross-reactivity of the primers, we would expect that primer cross-reactivity with non-target sequences in clinical samples would diminish compared to unmodified primers. Figure 4 indeed demonstrates that addition of 10 or 100 ng of human genomic DNA to the assay hampered accurate qPCR efficiency determinations for unmodified primers, whereas 5′-*o*-TINA modified primers were left unaffected. For non-spiked samples, 200 nM of unmodified primers at a T*a* of 60.0°C resulted in a qPCR efficiency of 100%, whereas samples spiked with human genomic DNA resulted in loss of target dilution linearity (qPCR efficiencies far above 100% in combination with very low coefficient of determination (R^2^) for data used for efficiency curve determinations) and loss of melting curve uniformity at especially lower target concentrations for the unmodified primers, which would normally lead to exclusion of the data before efficiency curve determinations (Figure 4). However, we have included these data to show the difference in qPCR efficiency and robustness between unmodified and 5′-*o*-TINA modified primers in samples spiked with human genomic DNA. In contrast, 200 nM of 5′-*o*-TINA modified primers at a Ta of 66.0°C spiked with equal amounts of human genomic DNA only lead to a minor parallel shift in Cq of the target dilution series, due to an increased background and thereby threshold of the assay. Furthermore, only minor changes in the melting curve profiles were observed (Figure 4). To verify the results, we replicated the experiment, spiking the samples with genomic DNA purified from *Escherichia coli* and observed similar results (Figure S3). The non-target cross-reactivity of the unmodified primers could be eliminated by increasing C*primers* to 400 nM and T*a* to 66.0°C (Figure S3).

### Prolongation of Annealing Times Changed the qPCR Efficiency Equally for Unmodified and 5′-*o*-TINA Modified Primers

In the design of the present study, we performed the qPCR assays utilizing a relatively short annealing time to increase the stringency of the assay. As expected, prolongation of the annealing time, while keeping the T*a* and C*primers* constant, increased the qPCR efficiency, but at the cost of assay stringency (Figure S4). The influence of annealing time on stringency was observed to be equal for both unmodified and 5′-*o*-TINA modified primers, as expected (Figure S4).

### The Unmodified qPCR Primers Showed Equal Limiting Effect on Cq, whereas for 5′-*o*-TINA Modified Primers the Reverse Primer Limited Cq

The limiting effect on Cq by different primer concentrations was evaluated by primer “chessboard titration” (Table S4). For unmodified primers, no significant difference in Cq was observed when equal concentrations of forward and reverse primer were limiting the qPCR assay. For 5′-*o*-TINA modified primers the reverse primer was the limiting primer (Table S4). To ease the comparison of different primer concentrations throughout the study, we used equal concentrations of forward and reverse primers in all other experiments.

### 5′-*o*-TINA Modified Primers Significantly Reduced the Optimal PCR Program Length in Octaplex End-point PCR

**Figure 5:**
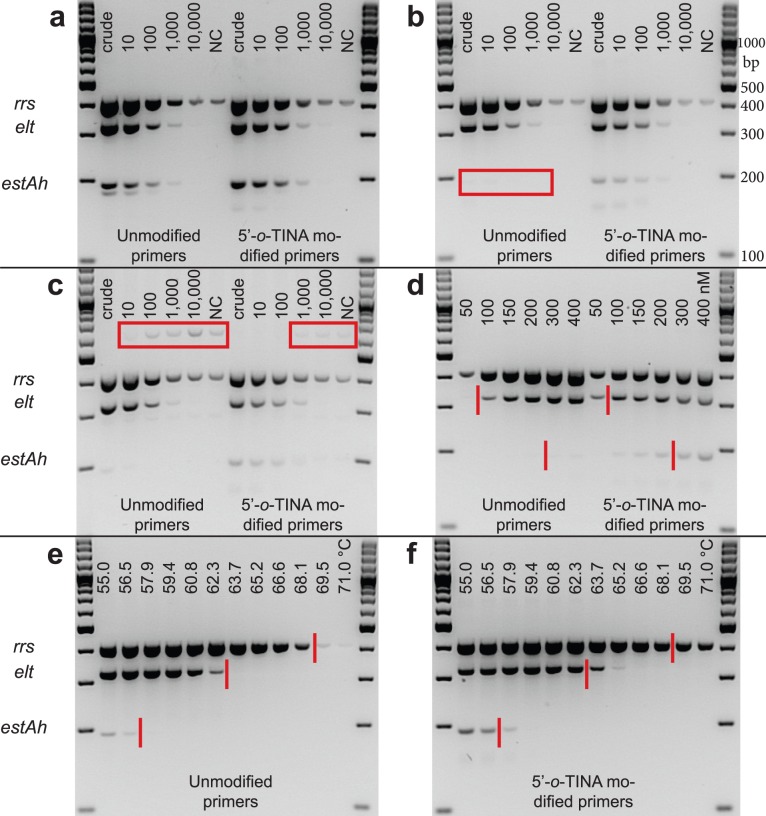
Amplification of an octaplex end-point PCR by unmodified primers and 5′-*o*-TINA modified primers. Data are shown for the D2168 strain entailing the *rrs* (401 bp), *elt* (322 bp) and *estAh* (190 bp) genes. (a) PCR program length of approximately 130 minutes, with a 10-fold target dilution series on crude bacterial lysate to 10,000-fold dilution and a negative control (NC) for unmodified primers and 5′-*o*-TINA modified primers. (b) Set-up identical to (a), but with a PCR program length of approximately 70 minutes. The red box highlights the lack of amplicons for the *estAh* gene with unmodified primers. (c) Set-up identical to (b), but additionally spiked with one µg of human genomic DNA per well. Red boxes highlight a non-specific amplicon with a size equaling the *ipaH* product. (d) 10-fold dilution of strain D2168 with different primer concentrations (C*primers*) of 50, 100, 150, 200, 300 and 400 nM for unmodified primers and 5′-*o*-TINA modified primers. Red lines are placed at the minimum C*primers* for unmodified primers. (e, f) Increasing annealing temperatures (T*a*) of 55.0, 56.6, 57.9, 59.4, 60.8, 62.3, 63.7, 65.2, 66.6, 68.1, 69.5 and 71.0°C on crude bacterial lysate for unmodified primers (e) and 5′-*o*-TINA modified primers (f). Red lines are placed at the maximum T*a* for unmodified primers. In (a-c, e, f), a C*primers* of 200 nM was used for each primer, except the *estAh* primers for which 400 nM of each primer was used. The PCR program with a length of approximately 70 minutes was used in (b-f).

As seen in Figure 5a, the unmodified primers and 5′-*o*-TINA modified primers amplified the three specific targets in the D2168 strain with equal analytical sensitivity using the published PCR program from Brandal LT et al. [18]. For both the unmodified and 5′-*o*-TINA modified primers an amplification product for the *rrs* internal control gene was amplified in the negative control and the unmodified PCR primers contrary to the 5′-*o*-TINA modified primers also amplified a non-specific product with a mass below the *estAh* amplification product in the two highest target concentrations. As the PCR program length was shortened by 60 minutes from a total PCR program length of approximately 130 minutes to approximately 70 minutes, all three specific amplicons could still be observed with similar analytical sensitivity for the 5′-*o*-TINA modified primers, whereas an *estAh* amplification product could not be detected by the unmodified primers (red box in Figure 5b). Using the faster PCR program, amplification of the *rrs* internal control gene in the negative controls was still observed. To ensure that both PCR programs worked equally well for the remaining five targets, we tested the remaining seven bacterial strains. The D2259 strain, which entail the *estAh* target confirmed the observations for the D2168 strain, whereas the remaining strains showed no difference in amplification, analytical sensitivity or non-specific amplification between unmodified and 5′-*o*-TINA modified primers connected to the length of the PCR program (Figure S5). For all strains and both unmodified primers and 5′-*o*-TINA modified primers, the *rrs* internal control gene was amplified in the negative control. We therefore changed the PCR buffer to our in-house Euro-Optima buffer system and for all eight bacterial strains the negative controls became negative. Unfortunately the Euro-Optima buffer system gave rise to a non-specific product for the unmodified primers with a mass equaling the mass of the *stx2* amplification product in strain 55989, D2259, D2260 and D3522 (Figure S5). In the remaining experiments, we continued to use the Qiagen Multiplex PCR Master Mix containing traces of genomic *E. coli* DNA, but not the virulence factors.

### 5′-*o*-TINA Modified Primers Increased the Robustness of the Octaplex End-point PCR on Spiked Samples

Four strains (fr1368, 55989, D2168 and D2188) collectively containing all eight targets were spiked with one µg of human genomic DNA isolated from the leukocyte fraction of whole blood. As seen in Figure 5c for strain D2168, all specific targets were amplified with a tendency towards lower analytical sensitivity for both unmodified primers and 5′-*o*-TINA modified primers. For all four bacterial strains, the spiking induced the amplification of a non-specific product with a size equaling the *ipaH* product and the non-specific product was marked in the spiked negative controls and highly diluted spiked bacterial strains (red boxes in Figure S6). Especially the unmodified DNA primers lead to the formation of the non-specific product, which could be detected at 10-fold to 100-fold higher bacterial target concentrations compared to the 5′-*o*-TINA modified primers (red boxes in Figure 5c and Figure S6). Based on these results we conclude that 5′-*o*-TINA modified primers were less prone to off-target amplification compared to unmodified primers, increasing the robustness for amplification of spiked targets in the octaplex end-point PCR.

### 5′-*o*-TINA Modified Primers Amplified the Octaplex End-point PCR at Reduced Primer Concentrations and Increased Annealing Temperatures

As for the *N. gonorrhoeae* qPCR assay, we compared the effect of lower C*primers* and increasing T*a* for unmodified primers and 5′-*o*-TINA modified primers in the octaplex end-point PCR assay with subsequent gel electrophoresis. Figure 5d illustrates the difference in detection of PCR products between unmodified and 5′-*o*-TINA modified primers at lower C*primers* for the *elt, estAh* and *rrs* genes in D2168. For the *elt*, *estAh*, *rrs*, partly *stx1* (D2188) and *stx2* targets, we observed a PCR product at half the C*primers* with 5′-*o*-TINA modified primers compared to unmodified primers, whereas we for the *aggR*, *eae*, *ipaH* and partly *stx1* (D3522) targets observed a stronger band at the lowest C*primers* that formed PCR products implying that 5′-*o*-TINA modified primers allow amplification at lower C*primers* compared to unmodified primers (Figure S7). Figure 5e and 5f shows the amplification by unmodified primers and 5′-*o*-TINA modified primers, respectively, on a temperature gradient for strain D2168 entailing the *elt*, *estAh* and *rrs* target genes. For all three targets 5′-*o*-TINA modified primers allowed visual detection of target amplification at T*a* increased by 1.5°C to above 3.0°C compared to amplification by unmodified primers. Similar results were obtained for T*a* evaluation for strain fr1368, 55989 and D2188 collectively, covering the remaining six targets when 5′-*o*-TINA modified primers were compared to unmodified primers. Thus, 5′-*o*-TINA modified primers increased the T*a* for all targets in the octaplex end-point PCR compared to unmodified primers (Figure S8).

## Discussion

In establishing a qPCR assay, the optimal balance between assay component concentrations and cycling conditions must be identified. In general, a trade-off must be made between sensitivity and specificity - high primer concentrations and low annealing temperature will maximize analytical sensitivity and qPCR efficiency at the cost of cross-reactivity and specificity and vice versa. In any qPCR system, a threshold can be found, where further reduction in primer concentration or further increase in annealing temperature will result in a reduction in PCR efficiency. In this study, we demonstrate the effect of an *o*-TINA modification on the 5′- position of the qPCR primers. In our model qPCR system, the 5′-*o*-TINA modification enables the use of half the primer concentration and simultaneously allowed for an additional increase in annealing temperature (of 3°C) compared to unmodified primers without reducing the qPCR efficiency. The present findings are likely to be generic to all 5′-*o*-TINA modified primers as they were verified for all 16 primers amplifying eight targets in an octaplex end-point PCR and are in concordance with our previous findings that *o*-TINA modifications at the 5′- and 3′- terminal ends of an oligonucleotide increase the melting point (Tm) and the analytical sensitivity of a hybridization capture assay significantly [6].

In the present study, we find that single nucleotide mismatches in the primer sequences alter Cq equally for unmodified and 5′-*o*-TINA modified primers, when unmodified and 5′-*o*-TINA modified primers are compared at the most stressed reaction condition that allows for a 100% qPCR efficiency. This clearly indicates that the 5′-*o*-TINA modified primers do not compromise the specificity of the qPCR compared to unmodified primers. Similar results have previously been shown for LNA containing primers, whereas Zip Nucleic Acid (ZNA) containing primers have been found to diminish the discrimination of non-target DNA controls [14]. We have previously shown that TINA molecules diminish the change in Tm by single nucleotide mismatches, when they are placed directly adjacent to non-complementary nucleobase pairs [6], [21], but that TINA molecules have no influence on the change in melting point by single nucleotide mismatches, when they are not placed directly adjacent to non-complementary nucleobase pairs. We therefore propose that *o*-TINA molecules influence the stacking of the first couple of adjacent nucleobases in the primer sequence, but does not influence the overall hybridization and cross-reactivity of the primers, whereas LNA molecules alters the conformation of the nucleotide helix from a B-DNA to an A-DNA like conformation [22], and ZNA containing primers diminish the overall backbone repulsion depending on the area of the primer they cover [23].

As no structural data concerning the precise positioning of the *o*-TINA molecule within the DNA double helix is currently available, we hypothesize the following mechanism: Due to the weaker hydrogen binding, adenine and thymine rich regions of dsDNA are more likely to be accessible for hybridization compared to guanine and cytosine rich regions. This fact in combination with the fact that the TINA molecules seem to intercalate equally well in-between all nucleobases, make us believe that *o*-TINA modified primers may be especially beneficial in the less stable adenine and thymine rich regions. Due to the terminal position, the pyrene moiety of the 5′-*o*-TINA molecule is likely to be stacking onto the terminal nucleobase pair with the phenyl moiety shifted towards the major groove and phosphate backbone of the oligonucleotide. This is supported by structure models and in-house unpublished PCR data.

As 5′-*o*-TINA modified primers allow for higher annealing temperatures and lower primer concentrations, we observed an increase in qPCR as well as end-point PCR robustness for samples spiked with genomic DNA from human or bacteria. The observed effects may partly be due to the higher annealing temperatures, which diminish the likelihood of cross-reactivity between specific and non-specific targets and amplification of non-target sequences and subsequent changes in the melting curve profiles. But as the target dilution linearity was sustained even at low primer concentrations for 5′-*o*-TINA modified primers, it also demonstrated that the 5′-*o*-TINA modified primers are still annealing to their specific targets in the presence of substantial amounts of non-specific targets. This is in contrast to the unmodified primers, for which increasing amounts of non-specific target amplification was observed resulting in loss of target dilution linearity. Likewise, the unmodified primers in the octaplex end-point PCR showed higher tendency to cross-reactivity at especially lower target concentrations compared to 5′-*o*-TINA modified primers for both samples spiked with human genomic DNA and when the buffer-system was changed to lesser stringent reaction conditions. In both cases, cross-reactivity was exclusively observed for the lesser stressed primer pairs (either the *ipaH* or *stx2* primer pairs) and was distinctly stronger for unmodified primers compared to 5′-*o*-TINA modified primers. The observed improved robustness for 5′-*o*-TINA modified primers may especially be beneficial when amplifying DNA directly from complex clinical samples and in the optimization of multiplex qPCR assays, in which the robustness of a single qPCR reaction limits the overall stringency of the multiplex qPCR assay. In general, added robustness is a major benefit contributed by modifying DNA oligonucleotides with nucleic acid intercalating molecules, as oligonucleotide stability and hybridization power is significant improved.

For the octaplex end-point PCR targeting diarrhoeagenic *E. coli*, we have demonstrated that the present buffer system from Qiagen entails traces of genomic DNA from *E. coli* leading to amplification of the *rrs* internal control gene from *E. coli* in the negative controls. This is a well-known problem for polymerases produced in bacterial systems and adds in this case to the draw-backs of the current octaplex end-point PCR assay for detection of diarrhoeagenic *E. coli* [24]. Although the 5′-*o*-TINA modified primers improved efficiency of the eight individual targets in the octaplex PCR, we still observed very diverse analytical sensitivity, optimal primer concentrations, optimal annealing temperatures and tendency to non-specific amplification for the eight primer pairs. Even though the introduction of 5′-*o*-TINA modified primers solved the biggest clinical draw-back of the assay in terms of the very long PCR program length, the uniformity of the assay still needs to be improved. The lack of assay uniformity illustrates, why multiplex assays should be designed following a predefined set of parameters and not just compost of a number of preexisting singleplex assays.

We propose that the effects by 5′-*o*-TINA modification of primers observed in the present study are likely to be applicable to design of PCR assays in general, as the changes in PCR reaction conditions applied in this study and the resulting impact on PCR efficiency and robustness follows predictable and well-established rules within basic PCR assay design.

## Supporting Information

Figure S1 **The** ***Neisseria gonorrhoeae porA*** **pseudogene target sequence and primer sequences for the qPCR assay.**
(PDF)

Figure S2 **Effect of C** ***primers*** **on Cq in qPCR at incrementally increasing T** ***a*** **.**
(PDF)

Figure S3 **Unmodified and 5′-** ***o*** **-TINA modified primers spiked with genomic DNA.**
(PDF)

Figure S4 **qPCR efficiency curves for unmodified and 5′-** ***o*** **-TINA modified primers at different annealing times.**
(PDF)

Figure S5 **Effect of PCR program length and PCR buffer for eight strains of diarrheagenic** ***E. coli*** **.**
(PDF)

Figure S6 **End-point PCR on crude bacterial lysates spiked with one µg of human genomic DNA.**
(PDF)

Figure S7 **Effect of C** ***primers*** **on the amplification of the octaplex end-point PCR.**
(PDF)

Figure S8 **Effect of T** ***a*** **on the amplification of the octaplex end-point PCR.**
(PDF)

Table S1 **Oligonucleotide concentration verification and purity control for all qPCR primers.**
(PDF)

Table S2 **Effect on Cq of single nucleotide mismatches throughout the qPCR primers.**
(PDF)

Table S3 **Effect on Cq of single nucleotide mismatches in the 3′-end nucleotide of qPCR primers.**
(PDF)

Table S4 **“Chessboard titration” of C** ***primers*** **for unmodified and 5′-** ***o*** **-TINA modified qPCR primers.**
(PDF)
