# Supplementary material for: Improved Efficiency and Robustness in qPCR and Multiplex End-Point PCR by Twisted Intercalating Nucleic Acid Modified Primers
Source: PLoS One. 2012 Jun 6;7(6):e38451. doi: 10.1371/journal.pone.0038451 (PMC3368873; doi:10.1371/journal.pone.0038451)
Supplement: Figure S1 — The Neisseria gonorrhoeae porA pseudogene target sequence and primer sequences for the qPCR assay. (PDF) [file pone.0038451.s001.pdf]

```
TTGGGCGGCGGGCTGAAGGCTGTTTGGCAGCTCGAGCAAGACGTATCCGTTGCCGGCGGCGGCGCGACCCGTTGGG
GTAACAGGGAATCCTTTATCGGCTTGGCAGGCGAATTCGGCACGGCGCTCGCCGGTCGCGTTGCGAATCCGTTTGA
CGATGCCAGCAAAGCCATTGATCCTTGGGACAGCAATAATAATGTGGCTTCGCAATTGGGTATTTTCAAACGCCAC
GACGGTATGCCGGTTTCCGTGCGTTACGATTCCCCCGGATTTTCCGGTTTCAGCGGCAGCATTCAATTTGTTCCGA
                                              gtttcagcggcagcattca / zgtttcagcggcagcattca
GTCAAAACAGCAAGTCCGCCTATACGCCTGCTACTTTCACGCTGGAAAGTAATCAGATGAAACCAGTTCCGGCTGT
                                                     ccggaactggtttcatctg / zccggaactggtttcatctg
TGTCGGCAAGCCGGGGTCGGATGTGTATTATGCCGGTCTGAATTACAAAAATGGCGGCTTTTTCGGAAATTATGCC
CTTAAATATGCGAAACACGCCAATGAGGGGCATGATGCTTTCTTTTTGTTCTTGCTCGGCAGAGCGAGTGATACCG
ATCCATTGAAAAACCATCAGGTACACCGCCTGACGGGCGGCTATGGGGAAGGCGGCTTGAATCTCGCCTTGGCGGC
TCAGTTGGATTTGTCTGAAAATGCCGACAAAACCAAAAACAGTACGACCGAAATTGCCGCCACTGCTTCCTACCGC
TTCGGTAATACAGTCCCGCGCATCAGCTATGCCCATGGTTTCGACTTTGTCGAACGCAGTCAGAAACGCGAACATA
CCAGCTATGATCAAATCATCGCCGGTGTCGATTACGATTTTTCCAAGCGCACTTCCGCCATCATGTCTGCCGCTTG
GCTGAAACGAAATACCGGCATCGGCAACTACACTCAAATTAATGCCGCCTCCGTTGGTCTGCGCCACAAATTCTAA
```

Supplementary Figure S1. The *Neisseria gonorrhoeae porA* pseudogene nucleotide sequence based on *N. gonorrhoeae* NCCP11945 with indication of match primers (in green). GenBank sequence CP001050.1 from nucleotide 736237 to 737148 (912 nucleotides) equaling locus tag NGK 0907 and protein ID ACF29586.1. Presented in Geneious Pro v5.4.3. Primer sequences were based on Hjemmevoll, S.O., Olsen,M.E., Sollid,J.U., Haaheim,H., Unemo,M. and Skogen,V. (2006) A fast real-time polymerase chain reaction method for sensitive and specific detection of the Neisseria gonorrhoeae porA pseudogene. *J. Mol. Diagn.*, **8**, 574-581.
