# Supplementary material for: Improved Efficiency and Robustness in qPCR and Multiplex End-Point PCR by Twisted Intercalating Nucleic Acid Modified Primers
Source: PLoS One. 2012 Jun 6;7(6):e38451. doi: 10.1371/journal.pone.0038451 (PMC3368873; doi:10.1371/journal.pone.0038451)
Supplement: Figure S2 — Effect of C primers on Cq in qPCR at incrementally increasing T a . (PDF) [file pone.0038451.s002.pdf]

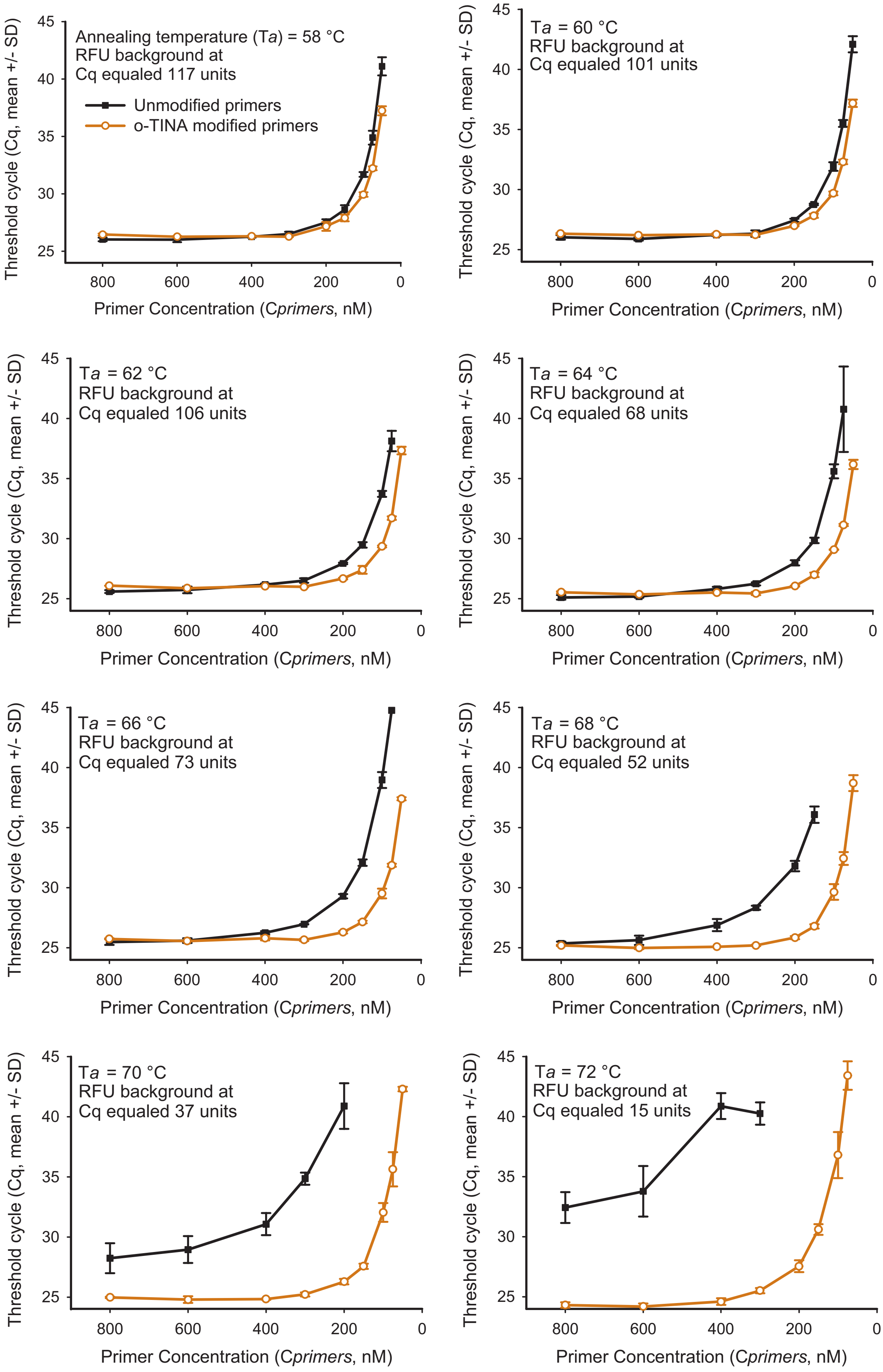

Supplementary Figure S2. Comparison of unmodified and 5’-*o*-TINA modified primer concentrations at different annealing temperatures (T*a*). The background relative fluorescence unit (RFU) at the Cq diminished as T*a* increased leading to lower optimal Cq levels.
