# Supplementary material for: Improved Efficiency and Robustness in qPCR and Multiplex End-Point PCR by Twisted Intercalating Nucleic Acid Modified Primers
Source: PLoS One. 2012 Jun 6;7(6):e38451. doi: 10.1371/journal.pone.0038451 (PMC3368873; doi:10.1371/journal.pone.0038451)
Supplement: Figure S3 — Unmodified and 5′- o -TINA modified primers spiked with genomic DNA. (PDF) [file pone.0038451.s003.pdf]

a

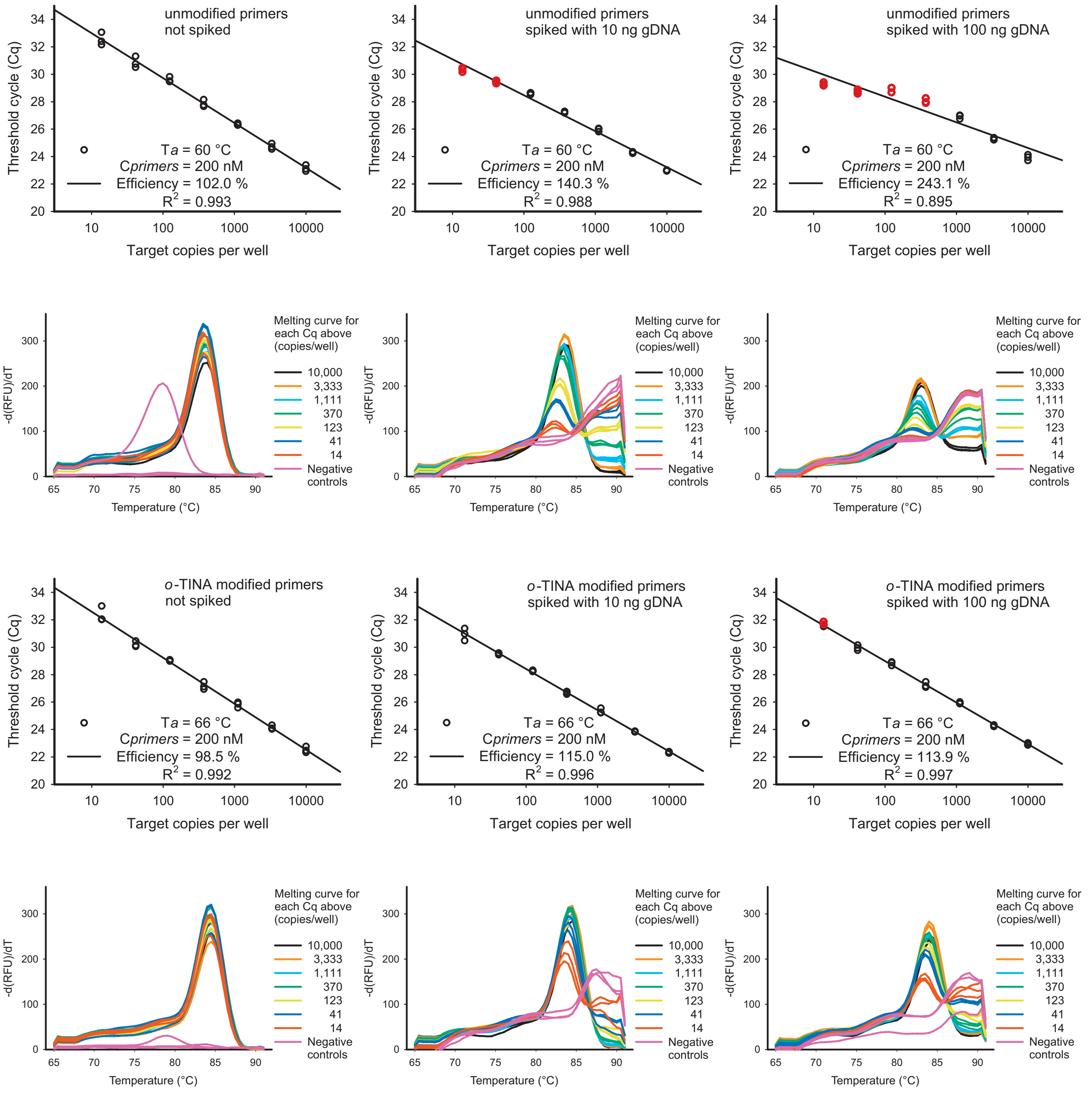

(Continues on next page)

b

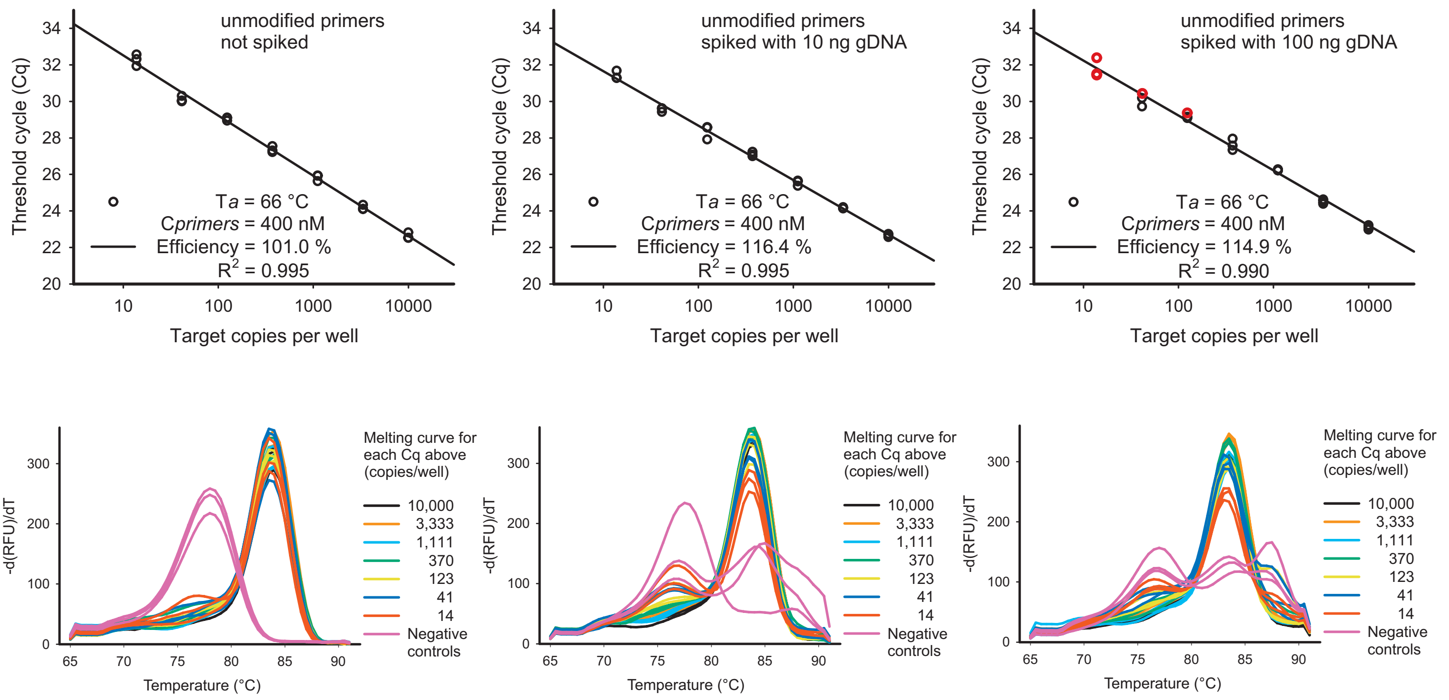

Supplementary Figure S3. Unmodified and 5'-*o*-TINA modified primers spiked with genomic DNA (gDNA). (a) Replication of experiment in Figure 4, but this time spiking was done with gDNA from *Escherichia coli*. The primer concentration (C*primers*) was 200 nM and the annealing temperatur (T*a*) was 60.0 °C for unmodified and 66.0 °C for 5'-*o*-TINA modified primers, respectively. (b) Unmodified primers spiked with human gDNA as in Figure 4, but with increased C*primers* of 400 nM and a T*a* of 66.0 °C. Each ***o*** on the efficiency curves represents one threshold cycle (Cq) determination on an amplification curve with a corresponding melting curve, reported as the first derivative. Cq determinations highlighted in red would normally have been excluded based on the amplification curve and melting curve evaluation.
