# Supplementary material for: Improved Efficiency and Robustness in qPCR and Multiplex End-Point PCR by Twisted Intercalating Nucleic Acid Modified Primers
Source: PLoS One. 2012 Jun 6;7(6):e38451. doi: 10.1371/journal.pone.0038451 (PMC3368873; doi:10.1371/journal.pone.0038451)
Supplement: Figure S4 — qPCR efficiency curves for unmodified and 5′- o -TINA modified primers at different annealing times. (PDF) [file pone.0038451.s004.pdf]

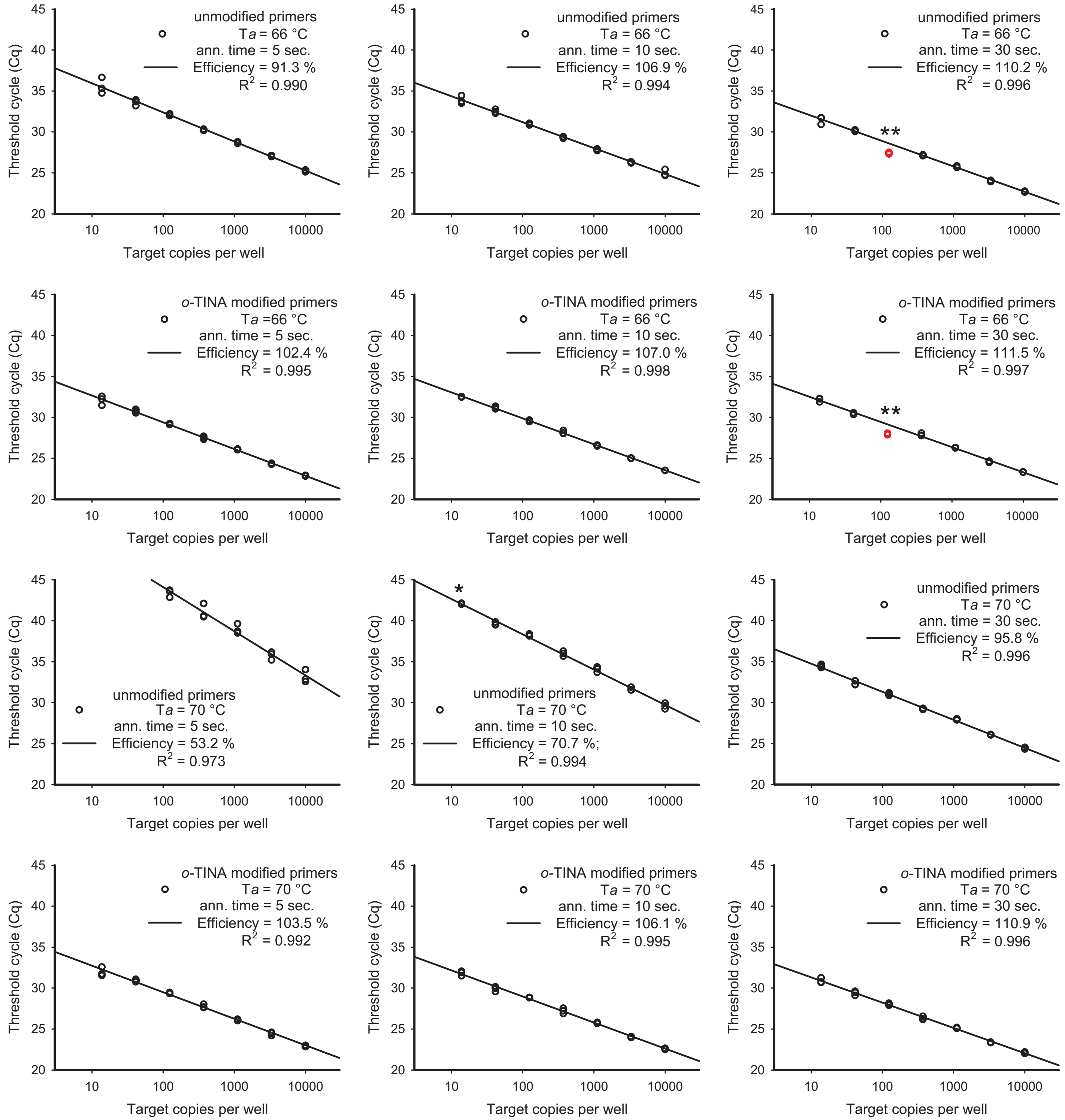

Supplementary Figure S4. Comparison of unmodified and 5'-*o*-TINA modified primer concentrations at three different annealing times and two different annealing temperatures (T*a*). Unmodified and 5'-*o*-TINA modified primers were compared on the same plate. *A single standard with 14 copies per well was excluded from the data analysis, as the Cq was 44.1 compared to a mean Cq of 42.1 with a SD of 0.1. **Both triplicate measurements were excluded for efficiency calculation, as the Cq's were approximately 1.5 cycle lower than expected (highlighted in red).

Eff. summary	Unmodified		5' *o*-TINA modified	
T*a* (°C); ann. time (seconds)	Efficiency (%)	R^2	Efficiency (%)	R^2
66; 5	91.3	0.990	102.4	0.995
66; 10	106.9	0.994	107.0	0.998
66; 30	110.2	0.996	111.5	0.997
70; 5	53.2	0.973	103.5	0.992
70; 10	70.7	0.994	106.1	0.995
70; 30	95.8	0.996	110.9	0.996
