# Supplementary material for: Improved Efficiency and Robustness in qPCR and Multiplex End-Point PCR by Twisted Intercalating Nucleic Acid Modified Primers
Source: PLoS One. 2012 Jun 6;7(6):e38451. doi: 10.1371/journal.pone.0038451 (PMC3368873; doi:10.1371/journal.pone.0038451)
Supplement: Figure S5 — Effect of PCR program length and PCR buffer for eight strains of diarrheagenic E. coli . (PDF) [file pone.0038451.s005.pdf]

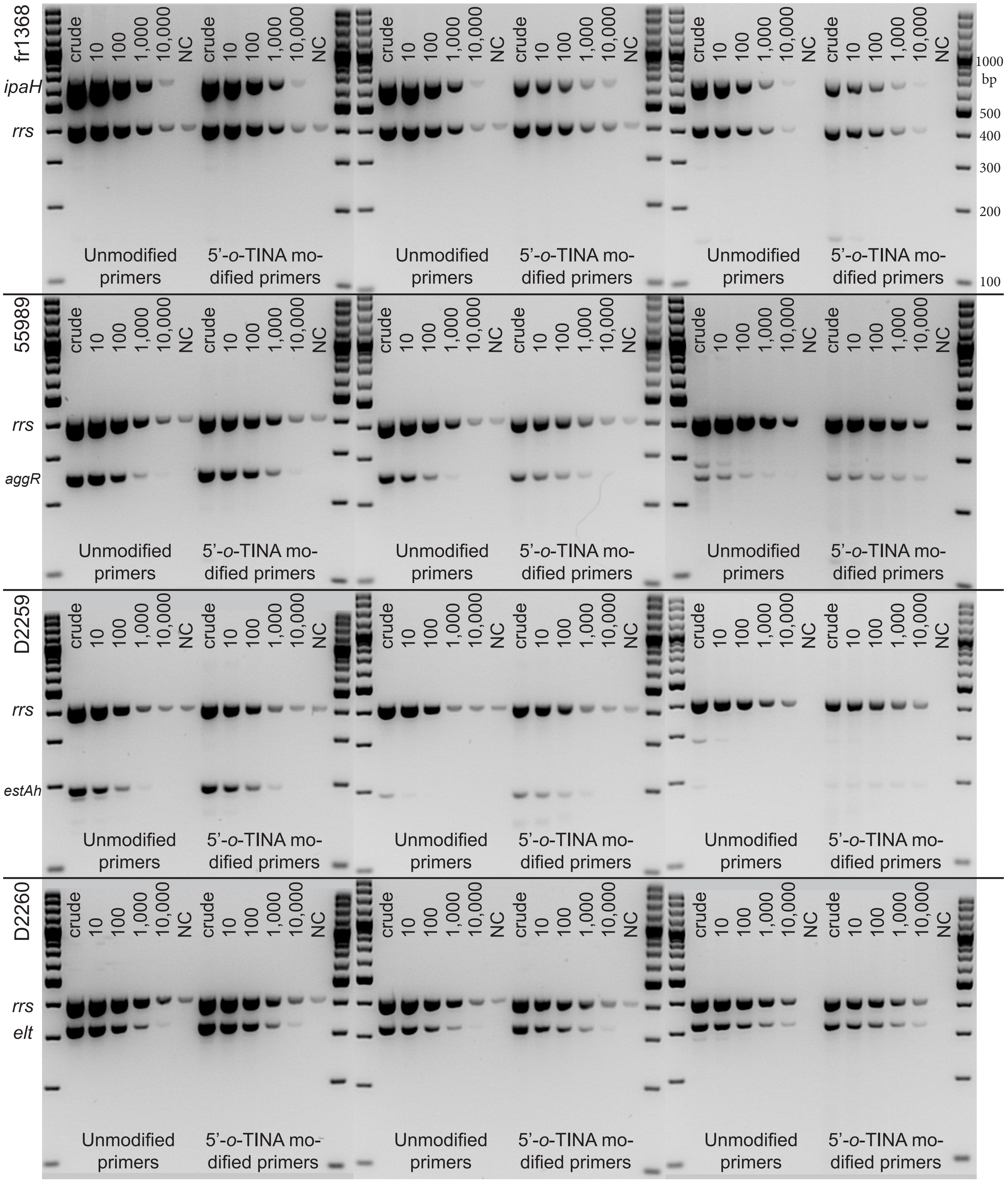

PCR program length: 130 minutes | 70 minutes | 70 minutes
PCR buffer: Qiagen buffer | Qiagen buffer | EuroOptima buffer

(Continues on next page)

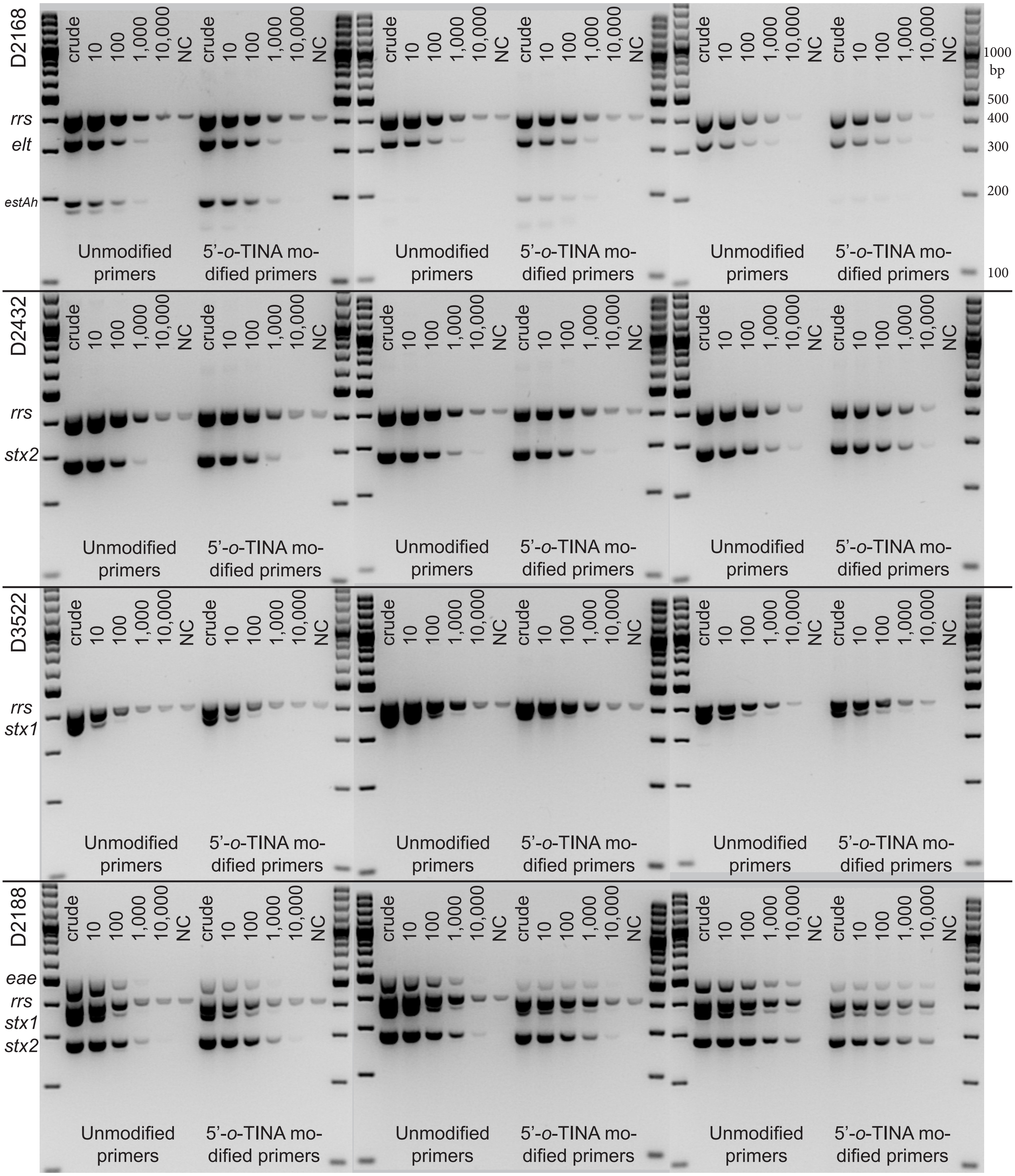

Supplementary Figure S5. Effect of different PCR program lengths and PCR buffer systems for eight strains of diarrheagenic *E. coli*. The left column shows the target amplification using the PCR program with a length of approximately 130 minutes in the Qiagen Multiplex PCR Master Mix. In the middle column, the PCR program length is reduced to approximately 70 minutes in the Qiagen buffer and in the right column the PCR program length is approximately 70 minutes in the EuroOptima buffer. C*primers* was 200 nM for each primer (double for *estAh* primers). Column separation: 100-bp DNA ladder.
