# Supplementary material for: Improved Efficiency and Robustness in qPCR and Multiplex End-Point PCR by Twisted Intercalating Nucleic Acid Modified Primers
Source: PLoS One. 2012 Jun 6;7(6):e38451. doi: 10.1371/journal.pone.0038451 (PMC3368873; doi:10.1371/journal.pone.0038451)
Supplement: Figure S6 — End-point PCR on crude bacterial lysates spiked with one µg of human genomic DNA. (PDF) [file pone.0038451.s006.pdf]

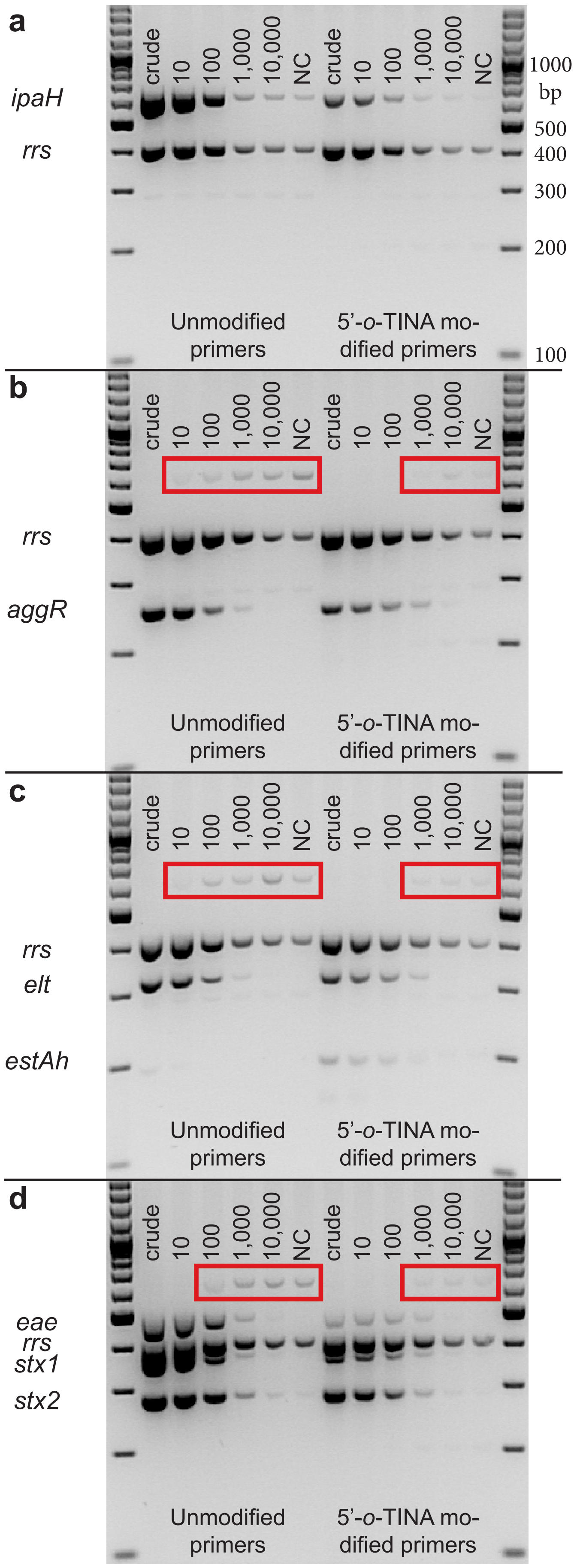

Supplementary Figure S6. Crude bacterial lysates spiked with one µg of human genomic DNA.

PCR program length of approximately 70 minutes, with a 10-fold target dilution series on crude bacterial lysate to 10,000-fold dilution and a negative control (NC) for unmodified primers and 5'-*o*-TINA modified primers.

Strain fr1368 in (a), strain 55989 in (b), strain D2168 in (c) and strain D2188 in (d). C*primers* was 200 nM for each primer (double for *estAh* primers). Marker: 100-bp DNA ladder.

A red box highlights the amplification of a non-specific product with a size equaling the *ipaH* product.
