# Supplementary material for: Improved Efficiency and Robustness in qPCR and Multiplex End-Point PCR by Twisted Intercalating Nucleic Acid Modified Primers
Source: PLoS One. 2012 Jun 6;7(6):e38451. doi: 10.1371/journal.pone.0038451 (PMC3368873; doi:10.1371/journal.pone.0038451)
Supplement: Figure S7 — Effect of C primers on the amplification of the octaplex end-point PCR. (PDF) [file pone.0038451.s007.pdf]

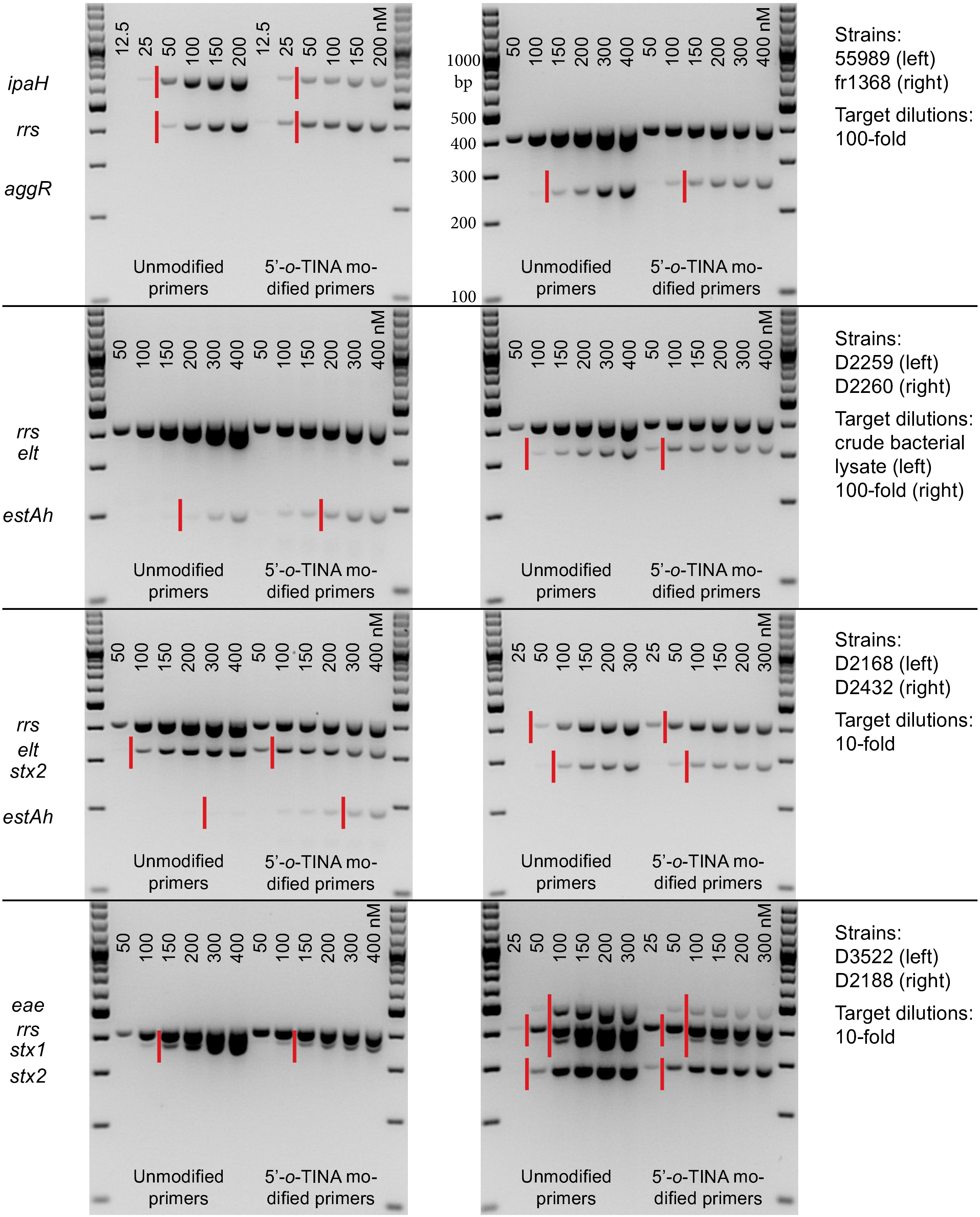

Supplementary Figure S7. Effect of different primer concentrations (C*primers*) on the amplification of eight strains of diarrheagenic *E. coli* by unmodified primers and 5'-*o*-TINA modified primers. Given C*primers* are double for the *estAh* primers. | is placed at the minimum C*primers* for unmodified primers.
