# Supplementary material for: Improved Efficiency and Robustness in qPCR and Multiplex End-Point PCR by Twisted Intercalating Nucleic Acid Modified Primers
Source: PLoS One. 2012 Jun 6;7(6):e38451. doi: 10.1371/journal.pone.0038451 (PMC3368873; doi:10.1371/journal.pone.0038451)
Supplement: Figure S8 — Effect of T a on the amplification of the octaplex end-point PCR. (PDF) [file pone.0038451.s008.pdf]

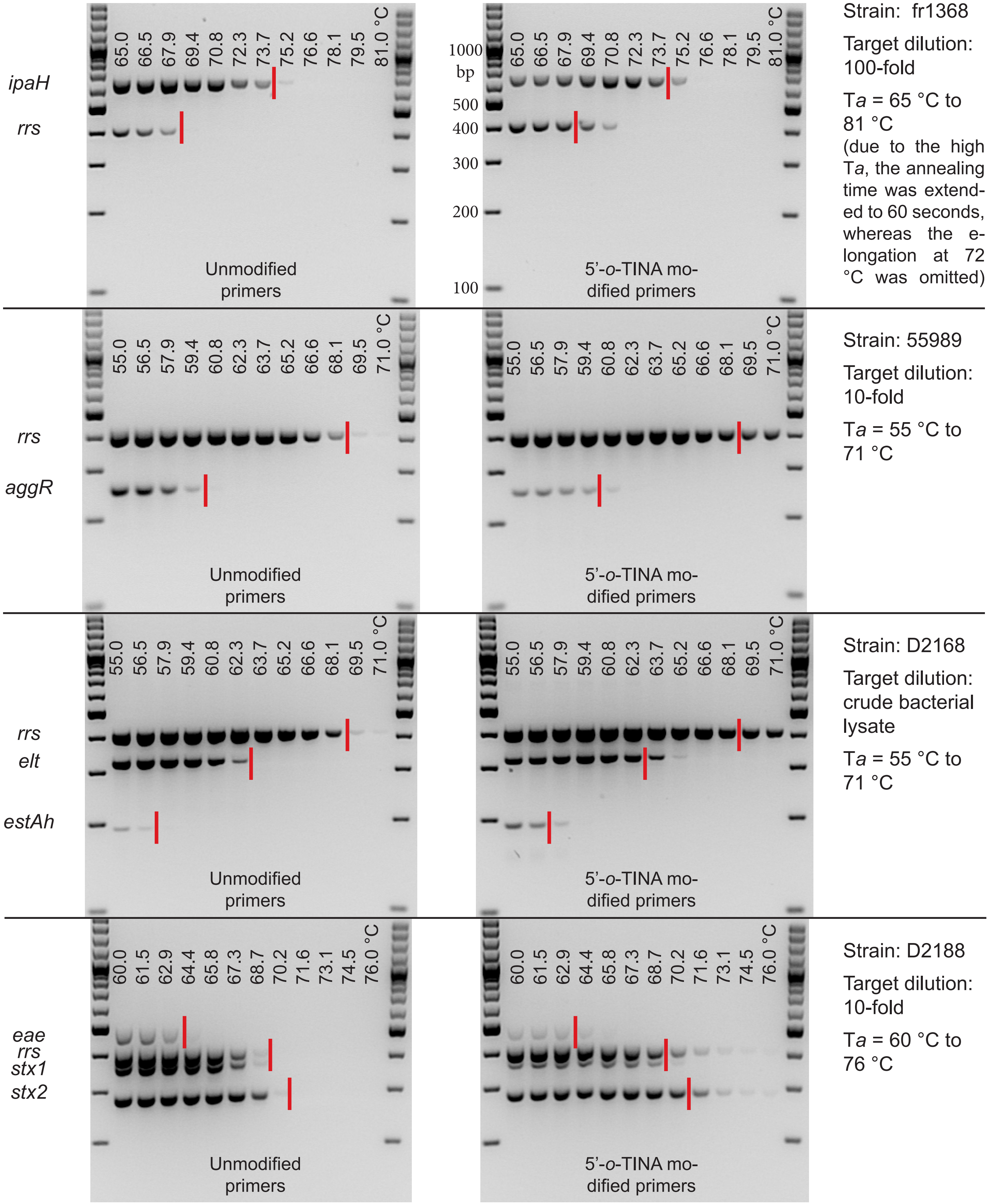

Supplementary Figure S8. Comparison of unmodified and 5'-*o*-TINA modified primers at different annealing temperatures (T*a*) for for four strains of diarrheagenic *E. coli* collectively covering all eight targets. C*primers* was 200 nM for each primer (double for *estAh* primers).
| placed at maximum T*a* for unmodified primers. Marker: 100-bp DNA ladder.
