# Supplementary material for: Improved Efficiency and Robustness in qPCR and Multiplex End-Point PCR by Twisted Intercalating Nucleic Acid Modified Primers
Source: PLoS One. 2012 Jun 6;7(6):e38451. doi: 10.1371/journal.pone.0038451 (PMC3368873; doi:10.1371/journal.pone.0038451)
Supplement: Table S1 — Oligonucleotide concentration verification and purity control for all qPCR primers. (PDF) [file pone.0038451.s009.pdf]

Supplementary Table S1. Oligonucleotide concentration verification and purity control for all qPCR primers.

		NanoDrop™ 1000 measurement		Liquid chromatography - mass spectrometry (LC-MS)				
Sequence (5' to 3')	Calculated Mass (Da)	Concentration (µM)	Difference in percent		Retention time (minutes)	LC/MS area percent	Intensity	Base peak mass (Da)
CCGGAACTGGTTTCATCTG	5794.8	125.4	0.47	**Peak 1**	**10.48**	**98.24**	**51500.0**	**5794.3**
				Peak 2	11.30	0.22	463.0	7440.3
				Peak 3	12.43	1.54	59.3	5718.8
GTTTCAGCGGCAGCATTCA	5803.8	95.7	0.10	Peak 1	7.65	0.12	69.4	6905.8
				Peak 2	8.86	0.12	114.0	3741.2
				**Peak 3**	**10.46**	**95.63**	**56800.0**	**5803.4**
				Peak 4	12.75	0.89	95.6	5677.7
C**T**GGAACTGGTTTCATCTG	5809.8	92.1	0.57	Peak 1	9.33	0.18	189.0	4349.1
				**Peak 2**	**10.24**	**99.12**	**77800.0**	**5809.3**
				Peak 3	12.50	0.70	23.4	5733.6
G**C**TTCAGCGGCAGCATTCA	5788.8	96.1	0.45	**Peak 1**	**10.44**	**98.93**	**69000.0**	**5788.7**
				Peak 2	12.62	1.07	191.0	5662.4
CC**A**GAACTGGTTTCATCTG	5778.8	110.1	0.73	Peak 1	9.53	0.22	251.0	4318.4
				**Peak 2**	**10.44**	**99.05**	**62500.0**	**5778.2**
				Peak 3	12.69	0.73	20.7	5702.1
GT**C**TCAGCGGCAGCATTCA	5788.8	97.6	1.57	Peak 1	7.69	0.12	53.8	6905.8
				Peak 2	8.79	0.14	135.0	3726.7
				**Peak 3**	**10.46**	**98.59**	**62500.0**	**5788.5**
				Peak 4	12.68	1.15	101.0	5662.7
CCGGAAC**C**GGTTTCATCTG	5779.8	98.2	0.82	Peak 1	9.36	0.19	231.0	4319.4
				**Peak 2**	**10.35**	**98.98**	**62500.0**	**5779.6**
				Peak 3	12.57	0.84	29.4	7606.6
GTTTCAG**T**GGCAGCATTCA	5818.8	92.4	1.31	Peak 1	7.79	0.13	79.6	7062.7
				Peak 2	8.96	0.20	144.0	3755.8
				**Peak 3**	**10.41**	**98.59**	**72200.0**	**5818.3**
				Peak 4	12.52	1.08	134.0	5692.5
CCGGAACT**A**GTTTCATCTG	5778.8	95.0	0.76	Peak 1	9.43	0.31	235.0	4318.3
				**Peak 2**	**10.29**	**99.08**	**55100.0**	**5778.6**
				Peak 3	12.56	0.61	24.7	7603.0
GTTTCAGC**A**GCAGCATTCA	5787.8	101.3	2.06	Peak 1	7.86	0.12	77.9	6982.7
				Peak 2	9.06	0.19	135.0	3724.8
				**Peak 3**	**10.57**	**98.73**	**73300.0**	**5787.5**
				Peak 4	12.53	0.96	91.3	5661.8
CCGGAACTGGTTT**G**ATCTG	5834.8	86.9	2.06	Peak 1	9.06	0.13	216.0	4045.0
				**Peak 2**	**10.21**	**99.23**	**55900.0**	**5834.2**
				Peak 3	12.60	0.64	22.6	5758.7

(Continues on next page)

		NanoDrop™ 1000 measurement		Liquid chromatography - mass spectrometry (LC-MS)				
Sequence (5' to 3')	Calcula-ted Mass (Da)	Concen-tration (µM)	Diffe-rence in percent		Reten-tion time (minutes)	LC/MS area percent	Intensity	Base peak mass (Da)
GTTTCAGCGGCAG**T**ATTCA	5818.8	93.8	1.58	Peak 1	7.76	0.14	77.9	6942.5
				Peak 2	8.93	0.17	149.0	3741.0
				**Peak 3**	**10.53**	**98.53**	**68600.0**	**5818.4**
				Peak 4	12.72	1.16	144.0	5692.7
CCGGAACTGGTTTCATC**C**G	5779.8	97.6	0.82	Peak 1	9.36	0.36	535.0	4334.0
				**Peak 2**	**10.28**	**97.71**	**65600.0**	**5779.6**
				Peak 3	11.15	0.35	507.0	6236.4
				Peak 4	12.49	1.58	90.4	5191.6
GTTTCAGCGGCAGCATT**T**A	5818.8	129.1	0.51	Peak 1	7.76	0.14	211.0	2809.2
				Peak 2	9.00	0.23	324.0	3741.0
				Peak 3	9.56	0.17	349.0	4359.3
				**Peak 4**	**10.60**	**96.61**	**85300.0**	**5818.8**
				Peak 5	12.90	2.84	310.0	5692.6
CCGGAACTGGTTTCATCT**A**	5778.8	134.0	0.82	Peak 1	9.40	0.21	413.0	4334.4
				**Peak 2**	**10.42**	**97.97**	**81400.0**	**5778.6**
				Peak 3	12.67	1.13	78.9	6957.5
				Peak 4	13.04	0.69	64.0	5809.2
GTTTCAGCGGCAGCATTC**G**	5819.8	120.3	0.71	Peak 1	6.12	0.14	59.8	7446.5
				Peak 2	7.19	0.15	173.0	7441.0
				Peak 3	7.76	0.17	187.0	2809.1
				Peak 4	9.03	0.27	264.0	3740.9
				Peak 5	9.56	0.25	343.0	4359.2
				**Peak 6**	**10.51**	**98.14**	**56100.0**	**5819.1**
				Peak 7	12.75	0.88	102.0	5743.8
CCGGAACTGGTTTCATCT**T**	5769.8	118.8	0.29	Peak 1	9.46	0.49	577.0	4334.2
				**Peak 2**	**10.43**	**98.65**	**76300.0**	**5769.5**
				Peak 3	12.70	0.45	20.8	6987.1
				Peak 4	13.00	0.41	41.9	5701.6
GTTTCAGCGGCAGCATTC**T**	5794.8	117.5	0.53	Peak 1	7.29	0.15	157.0	7441.0
				Peak 2	7.76	0.19	186.0	2809.1
				Peak 3	9.00	0.16	300.0	3741.1
				Peak 4	9.23	0.14	174.0	4029.9
				Peak 5	9.56	0.20	394.0	4358.7
				**Peak 6**	**10.47**	**98.49**	**90500.0**	**5794.3**
				Peak 7	12.91	0.68	56.8	5718.7
CCGGAACTGGTTTCATCT**C**	5754.8	118.3	0.57	Peak 1	9.53	0.38	532.0	4334.0
				**Peak 2**	**10.40**	**96.75**	**70200.0**	**5754.0**
				Peak 3	12.70	2.88	322.0	1735.6

(Continues on next page)

		NanoDrop™ 1000 measurement		Liquid chromatography - mass spectrometry (LC-MS)				
Sequence (5’ to 3’)	Calcula-ted Mass (Da)	Concen-tration (μM)	Diffe-rence in percent		Reten-tion time (minutes)	LC/MS area percent	Intensity	Base peak mass (Da)
GTTTCAGCGGCAGCATTCC	5779.8	121.8	1.13	Peak 1	9.03	0.18	280.0	3741.0
				Peak 2	9.56	0.21	399.0	4359.2
				**Peak 3**	**10.41**	**97.15**	**67300.0**	**5779.2**
				Peak 4	12.71	2.46	396.0	5665.4
**Z**CCGGAACTGGTTTCATCTG	6263.2	81.1	0.38	Peak 1	6.35	0.23	94.5	7449.5
				Peak 2	6.52	0.37	81.4	7448.9
				**Peak 3**	**9.92**	**99.40**	**50000.0**	**6262.6**
**Z**GTTTCAGCGGCAGCATTCA	6272.2	81.9	0.34	Peak 1	6.62	0.61	67.2	5554.0
				**Peak 2**	**9.62**	**99.39**	**38300.0**	**6271.8**
**Z**C**T**GGAACTGGTTTCATCTG	6278.2	108.2	0.48	Peak 1	5.28	0.18	299.0	2759.1
				Peak 2	5.45	0.26	248.0	3089.0
				Peak 3	5.99	0.35	569.0	4011.2
				Peak 4	6.32	1.32	364.0	4966.8
				Peak 5	6.52	0.75	346.0	5809.1
				**Peak 6**	**9.82**	**96.64**	**73700.0**	**6277.7**
				Peak 7	11.70	0.51	84.8	8269.3
**Z**G**C**TTCAGCGGCAGCATTCA	6257.2	90.4	0.35	Peak 1	6.29	0.19	150.0	4039.0
				Peak 2	6.86	0.98	270.0	5539.1
				**Peak 3**	**9.18**	**97.97**	**41500.0**	**6256.9**
				Peak 4	10.88	0.73	60.5	6189.3
				Peak 5	11.15	0.14	8.5	7272.4
**Z**CC**A**GAACTGGTTTCATCTG	6247.2	89.0	0.45	Peak 1	5.32	0.08	245.0	2759.3
				Peak 2	5.48	0.09	209.0	3088.7
				Peak 3	6.02	0.24	456.0	4011.0
				Peak 4	6.35	1.41	315.0	4966.8
				Peak 5	6.52	1.06	420.0	5777.9
				**Peak 6**	**9.95**	**96.79**	**43900.0**	**6246.8**
				Peak 7	11.73	0.33	18.1	8227.7
**Z**GT**C**TCAGCGGCAGCATTCA	6257.2	90.2	1.67	Peak 1	5.92	0.38	184.0	4039.0
				Peak 2	6.59	1.18	271.0	5539.4
				**Peak 3**	**9.69**	**97.71**	**38900.0**	**6257.0**
				Peak 4	11.50	0.73	41.9	8251.0
**Z**CCGGAAC**C**GGTTTCATCTG	6248.2	80.2	0.96	Peak 1	6.39	0.04	67.1	6162.4
				Peak 2	6.59	0.12	42.0	5933.4
				**Peak 3**	**9.52**	**99.84**	**31400.0**	**6247.7**

(Continues on next page)

		NanoDrop™ 1000 measurement		Liquid chromatography - mass spectrometry (LC-MS)				
Sequence (5’ to 3’)	Calcula-ted Mass (Da)	Concen-tration (µM)	Diffe-rence in percent		Reten-tion time (minutes)	LC/MS area percent	Intensity	Base peak mass (Da)
**Z**GTTTCAG**T**GGCAGCATTCA	6287.2	93.8	0.77	Peak 1	5.28	0.04	95.8	8287.4
				Peak 2	5.42	0.04	127.0	3091.3
				Peak 3	5.92	0.51	206.0	4054.1
				Peak 4	6.62	1.20	365.0	5569.4
				**Peak 5**	**9.59**	**97.23**	**41300.0**	**6286.8**
				Peak 6	11.15	0.23	79.8	6218.6
				Peak 7	11.42	0.75	35.0	8291.2
**Z**CCGGAACT**A**GTTTCATCTG	6247.2	95.4	1.37	Peak 1	5.32	0.09	188.0	2759.1
				Peak 2	5.48	0.06	211.0	3089.1
				Peak 3	6.09	0.25	274.0	3994.7
				Peak 4	6.55	2.16	370.0	5777.2
				**Peak 5**	**10.08**	**97.28**	**48900.0**	**6246.4**
				Peak 6	11.86	0.16	21.6	6170.7
**Z**GTTTCAGC**A**GCAGCATTCA	6256.2	95.4	2.02	Peak 1	5.92	0.39	196.0	3693.9
				Peak 2	6.62	1.27	425.0	5538.2
				**Peak 3**	**9.55**	**97.51**	**42200.0**	**6256.1**
				Peak 4	11.32	0.84	77.3	8250.2
**Z**CCGGAACTGGTTT**G**ATCTG	6303.2	88.0	2.15	Peak 1	5.45	0.06	96.9	7822.4
				Peak 2	6.02	0.08	193.0	4051.3
				Peak 3	6.35	0.86	79.9	7508.9
				**Peak 4**	**9.99**	**98.61**	**45700.0**	**6302.6**
				Peak 5	11.73	0.15	48.5	6226.0
**Z**GTTTCAGCGGCAG**T**ATTCA	6287.2	96.3	0.77	Peak 1	5.28	0.13	202.0	2777.1
				Peak 2	5.52	0.07	162.0	3107.0
				Peak 3	5.99	0.54	130.0	4054.1
				Peak 4	6.65	2.57	683.0	5818.3
				**Peak 5**	**9.61**	**95.59**	**42900.0**	**6286.7**
				Peak 6	11.14	0.40	108.0	6218.6
				Peak 7	11.41	0.70	45.4	8291.4
**Z**CCGGAACTGGTTTCATC**C**G	6248.2	110.8	0.86	Peak 1	5.99	0.05	171.0	3996.3
				Peak 2	6.32	0.40	97.7	8253.0
				Peak 3	8.03	0.22	26.7	8158.4
				**Peak 4**	**9.92**	**99.33**	**48600.0**	**6247.5**
**Z**GTTTCAGCGGCAGCATT**T**A	6287.2	106.5	2.30	Peak 1	6.69	0.27	154.0	5569.1
				**Peak 2**	**9.68**	**99.33**	**48300.0**	**6286.8**
				Peak 3	10.44	0.08	324.0	6154.3
				Peak 4	11.11	0.13	46.8	7995.6
				Peak 5	11.47	0.18	20.4	8294.8

(Continues on next page)

		NanoDrop™ 1000 measurement		Liquid chromatography - mass spectrometry (LC-MS)				
Sequence (5' to 3')	Calculated Mass (Da)	Concentration (µM)	Difference in percent		Retention time (minutes)	LC/MS area percent	Intensity	Base peak mass (Da)
**Z**CCGGAACTGGTTTCATCT**A**	6247.2	107.7	2.43	Peak 1	5.58	0.04	113.0	3072.4
				Peak 2	6.45	0.85	112.0	5777.1
				Peak 3	7.96	0.06	19.9	6771.6
				Peak 4	8.13	0.11	15.8	7429.1
				**Peak 5**	**9.97**	**98.67**	**62500.0**	**6246.7**
				Peak 6	11.99	0.27	45.9	7605.6
**Z**GTTTCAGCGGCAGCATTC**G**	6288.2	82.8	2.55	Peak 1	6.55	0.50	169.0	5569.7
				**Peak 2**	**9.59**	**99.50**	**38300.0**	**6288.3**
**Z**CCGGAACTGGTTTCATCT**T**	6238.2	105.0	1.44	Peak 1	6.45	0.32	103.0	3294.7
				Peak 2	9.03	0.34	62.4	7463.2
				**Peak 3**	**9.95**	**99.35**	**67100.0**	**6238.0**
**Z**GTTTCAGCGGCAGCATTC**T**	6263.2	108.4	0.90	Peak 1	6.65	0.20	118.0	5545.4
				**Peak 2**	**9.64**	**99.22**	**60400.0**	**6262.6**
				Peak 3	10.40	0.26	466.0	6130.2
**Z**CCGGAACTGGTTTCATCT**C**	6223.2	103.5	0.34	Peak 1	6.09	0.06	245.0	3971.3
				Peak 2	6.42	0.61	105.0	3284.9
				**Peak 3**	**10.01**	**99.32**	**69800.0**	**6222.6**
**Z**GTTTCAGCGGCAGCATTC**C**	6248.2	110.0	1.35	Peak 1	6.59	0.17	81.6	5530.2
				Peak 2	7.96	0.06	39.9	6266.1
				**Peak 3**	**9.71**	**99.61**	**50200.0**	**6247.6**
				Peak 4	10.44	0.16	384.0	6114.9

Supplementary Table S1. Verification of oligonucleotide concentrations by NanoDrop™ 1000 measurements and oligonucleotide purity by Liquid chromatography - mass spectrometry (LC-MS). Single nucleotide mismatches in the primer sequences are underlined and marked in **bold** blue, whereas 5'-*o*-TINA modifications are indicated by **Z**. Oligonucleotide concentrations (µM) were calculated as the mean absorbance measurement at 260 nm (A260 of three single measurements) x dilution factor (a stock concentration from the oligonucleotide supplier of 100 µM was used) x 33 divided by the calculated mass. The difference in percent for each oligonucleotide was calculated as the standard deviation (SD) divided by the mean absorbance measurement at 260 nm x 100. For each LC-MS analysis, the retention time (in minutes) for each peak is reported together with the area in percent of the peak in the LC-MS spectra, the intensity of the peak and the mass detected for each peak. LC-MS analyses were run on a Thermo LCQ Fleet in negative mode and calculations were done in Promass for Xcalibur. For LC-MS an ACE-3 C18-300, 50 x 2.1 mm column was used with the analytical conditions of 50 °C; A gradient from 0-D% B over 20 minutes, A being water; 10 µM EDTA; 1% HFIPA; 0.1% DIEA, B being water: acetonitrile (35:65); 10 µM EDTA; 1% HFIPA; 0.1% DIEA and D being 25 for unmodified or 50 for 5'-*o*-TINA modified primers.
