# Supplementary material for: Improved Efficiency and Robustness in qPCR and Multiplex End-Point PCR by Twisted Intercalating Nucleic Acid Modified Primers
Source: PLoS One. 2012 Jun 6;7(6):e38451. doi: 10.1371/journal.pone.0038451 (PMC3368873; doi:10.1371/journal.pone.0038451)
Supplement: Table S2 — Effect on Cq of single nucleotide mismatches throughout the qPCR primers. (PDF) [file pone.0038451.s010.pdf]

	150nM	200nM	300nM	400nM	600nM	800nM
FP 5'-C**T**GGAACTGGTTTCATCTG-3'<br>RP 5'-G**C**TTCAGCGGCAGCATTCA-3'	1.1	1.2	0.6	1.2	3.2	3.4
CC**A**GAACTGGTTTCATCTG<br>GT**C**TCAGCGGCAGCATTCA	0.7	1.2	0.9	1.8	4.8	7.2
CCGGAAC**C**GGTTTCATCTG<br>GTTTCAG**T**GGCAGCATTCA	-0.5	0.7	0.9	3.0	3.0	-0.2
CCGGAACT**A**GTTTCATCTG<br>GTTTCAGC**A**GCAGCATTCA	0.4	1.5	2.1	5.5	9.9	Negative
CCGGAACTGGTTT**G**ATCTG<br>GTTTCAGCGGCAG**T**ATTCA	0.0	1.3	1.2	3.4	3.9	2.6
CCGGAACTGGTTTCATC**C**G<br>GTTTCAGCGGCAGCATT**T**A	6.2	6.9	7.1	12.6	12.8	Negative
CCGGAACTGGTTTCATCT**A**<br>GTTTCAGCGGCAGCATTC**G**	9.7	8.7	9.6	15.5	Negative	Negative
Annealing temperature (T*a*)	60**	60 (62)	62 (64)	66* (68)	68 (70***)	70
Efficiency (%)	93.7	99.6<br>(91.9)	100.0<br>(97.8)	102.1<br>(98.2)	101.7<br>(83.9)	99.2
R^2	0.999	0.992<br>(0.991)	0.992<br>(0.990)	0.995<br>(0.993)	0.993<br>(0.973)	0.972
**Z**C**T**GGAACTGGTTTCATCTG<br>**Z**G**C**TTCAGCGGCAGCATTCA	-0.2	-0.1	0.1	2.1	0.5	1.3
**Z**CC**A**GAACTGGTTTCATCTG<br>**Z**GT**C**TCAGCGGCAGCATTCA	2.2	2.7	3.2	7.6	5.6	5.8
**Z**CCGGAAC**C**GGTTTCATCTG<br>**Z**GTTTCAG**T**GGCAGCATTCA	1.5	2.1	2.9	3.5	3.7	3.2
**Z**CCGGAACT**A**GTTTCATCTG<br>**Z**GTTTCAGC**A**GCAGCATTCA	-0.3	1.2	3.1	7.2	7.4	6.2
**Z**CCGGAACTGGTTT**G**ATCTG<br>**Z**GTTTCAGCGGCAG**T**ATTCA	-0.5	0.0	1.2	2.9	3.6	2.4
**Z**CCGGAACTGGTTTCATC**C**G<br>**Z**GTTTCAGCGGCAGCATT**T**A	4.7	6.7	8.3	11.0	11.9	10.1
**Z**CCGGAACTGGTTTCATCT**A**<br>**Z**GTTTCAGCGGCAGCATTC**G**	7.1	8.8	10.9	Negative	Negative	Negative
Annealing temperature (T*a*)	64 (66)	66 (68)	68 (70)	70	70*	70
Efficiency (%)	99.9<br>(94.2)	100.9<br>(96.1)	102.6<br>(99.1)	99.2	102.0	104.2
R^2	0.998<br>(0.997)	0.995<br>(0.992)	0.997<br>(0.994)	0.995	0.994	0.994

Supplementary Table S2. Change in Cq by single nucleotide mismatches (ΔCq) (underlined and marked in **bold** blue) in unmodified and 5'-*o*-TINA (**Z**) modified primer pairs at different primer concentrations (C*primers*) from 150 nM to 800 nM of each primer. FP is the forward primer and RP the reverse primer nucleotide sequences, respectively. "Negative" samples had major changes in melting curve profiles or no Cq could be determined at cycle 45. All measurements were done in triplicate and outliers were excluded (one * per exclusion). Experiments were performed at the highest annealing temperature (T*a*) that allowed for a qPCR efficiency of 100 % for the matching primer pair. For each matching primer pair, the qPCR efficiency and coefficient of determination (R^2) are presented together with the drop in qPCR efficiency, when T*a* was incrementally increased (presented in brackets).
