# Supplementary material for: Improved Efficiency and Robustness in qPCR and Multiplex End-Point PCR by Twisted Intercalating Nucleic Acid Modified Primers
Source: PLoS One. 2012 Jun 6;7(6):e38451. doi: 10.1371/journal.pone.0038451 (PMC3368873; doi:10.1371/journal.pone.0038451)
Supplement: Table S3 — Effect on Cq of single nucleotide mismatches in the 3′-end nucleotide of qPCR primers. (PDF) [file pone.0038451.s011.pdf]

Forward primer (FP) \ Reverse primer (RP)	GCTTCAGCG-GCAGCATTCA	GTTTCAGCG-GCAGCATTC**G**	GTTTCAGCG-GCAGCATTC**T**	GTTTCAGCG-GCAGCATTC**C**
CCGGAACTGGTTTCATCTG	27.2	30.8 (+3.6)	35.9 (+8.7)	42.5 (+15.3)
CCGGAACTGGTTTCATCT**A**	33.1 (+5.9)	38.0 (+10.7)	40.9 (+13.7)	40.7 (+13.4)
CCGGAACTGGTTTCATCT**T**	33.9 (+6.6)	38.0 (+10.8)	44.3 (+17.1)	38.3 (+11.1)
CCGGAACTGGTTTCATCT**C**	40.7 (+13.5)	Negative	Negative	Negative
Annealing temperature (T*a*, °C)	60			
Efficiency (%)	95.3			
R^2	0.995			
**Forward primer (FP) \ Reverse primer (RP)**	**Z**GCTTCAGCG-GCAGCATTCA	**Z**GTTTCAGCG-GCAGCATTC**G**	**Z**GTTTCAGCG-GCAGCATTC**T**	**Z**GTTTCAGCG-GCAGCATTC**C**
**Z**CCGGAACTGGTTTCATCTG	26.3	29.1 (+2.8)	33.3 (+6.9)	30.3 (+4.0)
**Z**CCGGAACTGGTTTCATCT**A**	32.2 (+5.9)	36.4 (+10.0)	37.2 (+10.9)	38.0 (+11.7)
**Z**CCGGAACTGGTTTCATCT**T**	33.1 (+6.8)	36.6 (+10.3)	41.7 (+15.4)	37.9 (+11.5)
**Z**CCGGAACTGGTTTCATCT**C**	39.9 (+13.6)	38.0 (+11.6)	Negative	Negative
Annealing temperature (T*a*, °C)	66			
Efficiency (%)	101.7			
R^2	0.995			

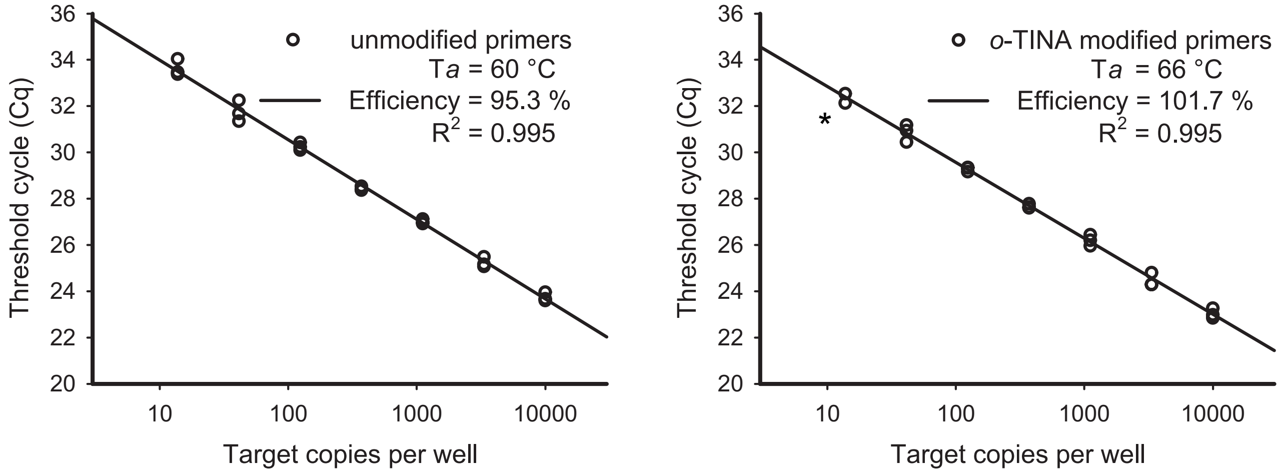

Supplementary Table S3. Change in Cq by single nucleotide mismatches (ΔCq) at the 3' position (underlined and marked in **bold** blue) in unmodified and 5'-*o*-TINA (**Z**) modified primer pairs. A primer concentration (C*primers*) of 200 nM was used with 1000 copies of target per well in triplicate measurements. For unmodified and 5'-*o*-TINA modified primers an annealing temperature (T*a*) of 60 °C and 66 °C was used, respectively. Data are presented as mean threshold cycle (Cq) with ΔCq in branckets. "Negative" samples had major changes in melting curve profiles or no Cq could be determined at cycle 45. The efficiency for the unmodified primers is below 100% in the present experiment, which increases the observed ΔCq. *A single standard with 14 copies per well was excluded from the data analysis for the 5'-*o*-TINA modified primers, as the Cq was significantly lower compared with the two other Cq determinations (31.4 compared to a mean Cq of 32.3 with a SD of 0.3).
