# Supplementary material for: Improved Efficiency and Robustness in qPCR and Multiplex End-Point PCR by Twisted Intercalating Nucleic Acid Modified Primers
Source: PLoS One. 2012 Jun 6;7(6):e38451. doi: 10.1371/journal.pone.0038451 (PMC3368873; doi:10.1371/journal.pone.0038451)
Supplement: Table S4 — “Chessboard titration” of C primers for unmodified and 5′- o -TINA modified qPCR primers. (PDF) [file pone.0038451.s012.pdf]

| **Unmodified primers** | *Cprimers* | | |
|---|---|---|---|
| Reverse primer (RP) / Forward primer (FP) | 100 nM | 200 nM | 400 nM |
| 100 nM | 37.8; 0.2 (+12.3) | 33.4; 1.0 (+7.8) | 31.8; 0.8 (+6.2) |
| 200 nM | 32.1; 0.4 (+6.6) | 28.7; 0.5 (+3.2) | 27.1; 0.1 (+1.6) |
| 400 nM | 29.8; 0.4 (+4.3) | 26.8; 0.3 (+1.2) | 25.5; 0.1 (0.0) |
| | | | |
| **5’-o-TINA modified primers** | | | |
| Reverse primer (RP) / Forward primer (FP) | 100 nM | 200 nM | 400 nM |
| 100 nM | 29.7; 0.1 (+3.9) | 26.7; 0.2 (+0.9) | 26.1; 0.1 (+0.3) |
| 200 nM | 28.6; 0.0 (+2.8) | 26.3; 0.1 (+0.5) | 25.3; 0.1 (-0.5) |
| 400 nM | 28.7; 0.2 (+2.9) | 26.0; 0.2 (+0.2) | 25.8; 0.1 (0.0) |
| Annealing temperature (T*a*, °C) | 66 | | |
| Efficiency (%) | 104.0 | | |
| $R^2$ | 0.995 | | |

**Supplementary Table S4.** “Chessboard titration” of unmodified and 5’-*o*-TINA modified primers. 1000 copies of target was used per well and threshold cycle (Cq) determinations were done as triplicate measurements. Results are reported as Cq with standard deviation (SD) and the change in Cq compared to the 400 nM primer concentration in brackets. The forward primer nucleotide sequence was 5’-CCG-GAACTGGTTTCATCTG-3’ and the reverse primer sequence was 5’-GTTTCAGCGGCAGCATTCA-3’. No significant changes in Cq by different primer concentrations were observed for the unmodified primers, whereas the reverse primer in the 5’-*o*-TINA modified primer pair was significantly limiting the qPCR assay. To ease the comparison of different primer concentrations, we used equal amounts of each primer in all other experiments.
